# Inositol hexakisphosphate is required for Integrator function

**DOI:** 10.1038/s41467-022-33506-3

**Published:** 2022-09-30

**Authors:** Min-Han Lin, Madeline K. Jensen, Nathan D. Elrod, Kai-Lieh Huang, Kevin A. Welle, Eric J. Wagner, Liang Tong

**Affiliations:** 1 Department of Biological Sciences, Columbia University, New York, NY 10027 USA; 2 Department of Biochemistry and Molecular Biology, The University of Texas Medical Branch, Galveston, TX 77550 USA; 3 Department of Biochemistry and Biophysics, Center for RNA Biology, University of Rochester School of Medicine and Dentistry, Rochester, NY 14642 USA; 4 Center for Advanced Research Technologies, University of Rochester School of Medicine and Dentistry, Rochester, NY 14642 USA

**Keywords:** Cryoelectron microscopy, Transcription

## Abstract

Integrator is a multi-subunit protein complex associated with RNA polymerase II (Pol II), with critical roles in noncoding RNA 3′-end processing and transcription attenuation of a broad collection of mRNAs. IntS11 is the endonuclease for RNA cleavage, as a part of the IntS4-IntS9-IntS11 Integrator cleavage module (ICM). Here we report a cryo-EM structure of the *Drosophila* ICM, at 2.74 Å resolution, revealing stable association of an inositol hexakisphosphate (IP_6_) molecule. The IP_6_ binding site is located in a highly electropositive pocket at an interface among all three subunits of ICM, 55 Å away from the IntS11 active site and generally conserved in other ICMs. We also confirmed IP_6_ association with the same site in human ICM. IP_6_ binding is not detected in ICM samples harboring mutations in this binding site. Such mutations or disruption of IP_6_ biosynthesis significantly reduced Integrator function in snRNA 3′-end processing and mRNA transcription attenuation. Our structural and functional studies reveal that IP_6_ is required for Integrator function in *Drosophila*, humans, and likely other organisms.

## Abstract (web summary)

Integrator is a multi-subunit protein complex with roles in noncoding RNA 3′-end processing and mRNA transcription attenuation. Here, the authors reveal a conserved binding site for IP_6_ in the Integrator cleavage module and show that IP_6_ binding is required for Integrator function.

## Introduction

Integrator is a 15-subunit complex associated with RNA polymerase II (Pol II) that is crucial for 3′-end processing of snRNAs^1^ and other noncoding RNAs^2–5^, as well as transcription attenuation through cleavage of a broad set of nascent mRNAs^6–10^. The broad importance of Integrator function to gene regulation is evidenced by the wide range of human disease states attributed to dysfunction of its subunits^11,12^.

The Integrator subunits, named IntS1 through IntS15, can be purified as a complex but also form several sub-modules. For example, our earlier studies have shown that IntS4-IntS9-IntS11 forms the Integrator cleavage module (ICM)^13,14^. IntS9 and IntS11 are paralogs of CPSF100 and CPSF73 in the canonical and U7 replication-dependent histone pre-mRNA 3′-end processing machineries, and CPSF73 catalyzes the cleavage reaction in both machineries^15–17^. The subunits IntS10-IntS13-IntS14 form a putative nucleic acid binding module^18,19^, and the IntS5-IntS8 complex^19,20^ is critical for recruiting protein phosphatase 2 A (PP2A)^20–22^.

The structures of several Integrator components have been reported over the years, including the C-terminal domain (CTD2) complex of human IntS9-IntS11^23^, the N- and C-terminal domains of human IntS3^24,25^, human IntS13-IntS14 complex^18^, and the human ICM^19^. In addition, the structures of human Integrator in complex with PP2A^21^ and Pol II^26,27^ were reported recently. These structures reveal how Integrator is organized overall. However, insight is still lacking as to how Integrator activity, especially that of the endonuclease IntS11^1^, is regulated within this machinery and by other factors.

Here we report a cryo-EM structure of the *Drosophila* ICM at 2.74 Å resolution, which unexpectedly reveals the stable association of an inositol hexakisphosphate (IP_6_) molecule. We have also identified IP_6_ binding to the same site in the human ICM. Mutations in the IP_6_ binding site disrupt IP_6_ binding but not Integrator assembly. On the other hand, such mutations or disruption of IP_6_ biosynthesis significantly reduce Integrator function in snRNA 3′-end processing and mRNA transcription attenuation.

## Results and discussion

### Structure of *Drosophila* ICM

**Fig. 1:**
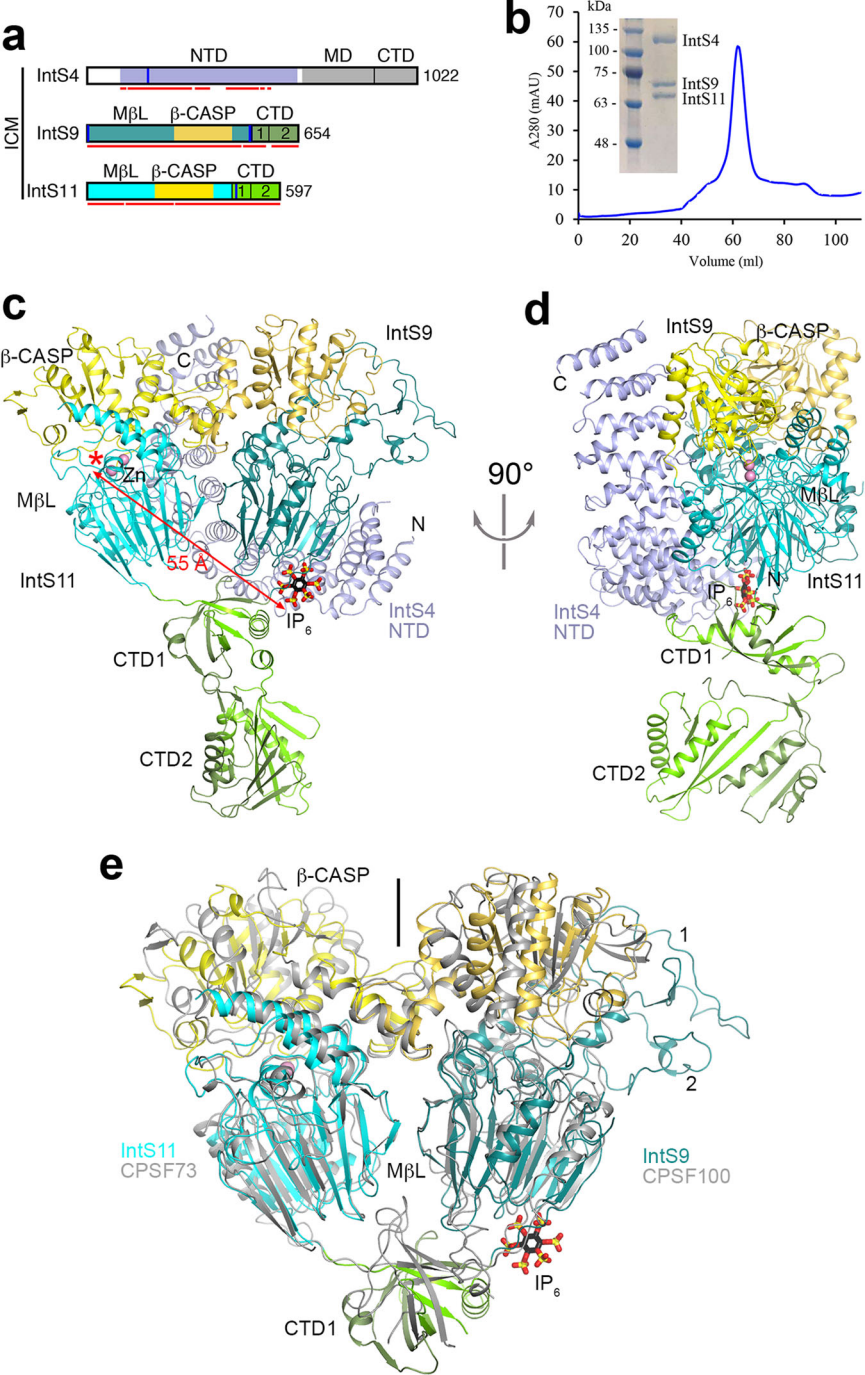
An unexpected IP_6_-binding site in the *Drosophila* IntS4-IntS9-IntS11 complex (ICM). **a** Domain organizations of *Drosophila* IntS4, IntS9, and IntS11. The domains are named and given different colors. The domains of IntS9 are shown in slightly darker colors compared to IntS11. Residues observed in the structure of the IntS4-IntS9-IntS11 complex are indicated with the red lines. The vertical bars in blue represent positively charged residues in the IP_6_ binding site. ICM Integrator cleavage module, NTD N-terminal domain, MD middle domain, CTD C-terminal domain, MβL metallo-β-lactamase, β-CASP metallo-β-lactamase-associated CPSF, Artemis, SNM1/PSO2. **b** Gel filtration profile of *Drosophila* ICM. Inset: SDS gel of the purified complex. The experiment was done at least three times with similar results. **c** The overall structure of the *Drosophila* ICM. The domains are colored as in panel a and labeled. The IP_6_ molecule is shown as stick models (black for carbon atoms). **d** Another view of the structure of the IntS4-IntS9-IntS11 complex, related to that of panel c by a 90° rotation around the vertical axis. **e** Overlay of the dimer of the metallo-β-lactamase and β-CASP domains of IntS9-IntS11 (in color) in the ICM with the dimer of the equivalent domains of human CPSF100-CPSF73 (gray) in the active histone pre-mRNA 3′-end processing machinery. The pseudo two-fold axis of this dimer is along the vertical direction (indicated with the black line). The CTD1s of the two structures assume very different positions, and the CTD2s are not shown. IntS9 metallo-β-lactamase domain contains two insertions, residues 34–83 (labeled 1) and 150–179 (2), that are positioned next to the β-CASP domain. The structure figures were produced with PyMOL (www.pymol.org) unless otherwise indicated.

**Fig. 2:**
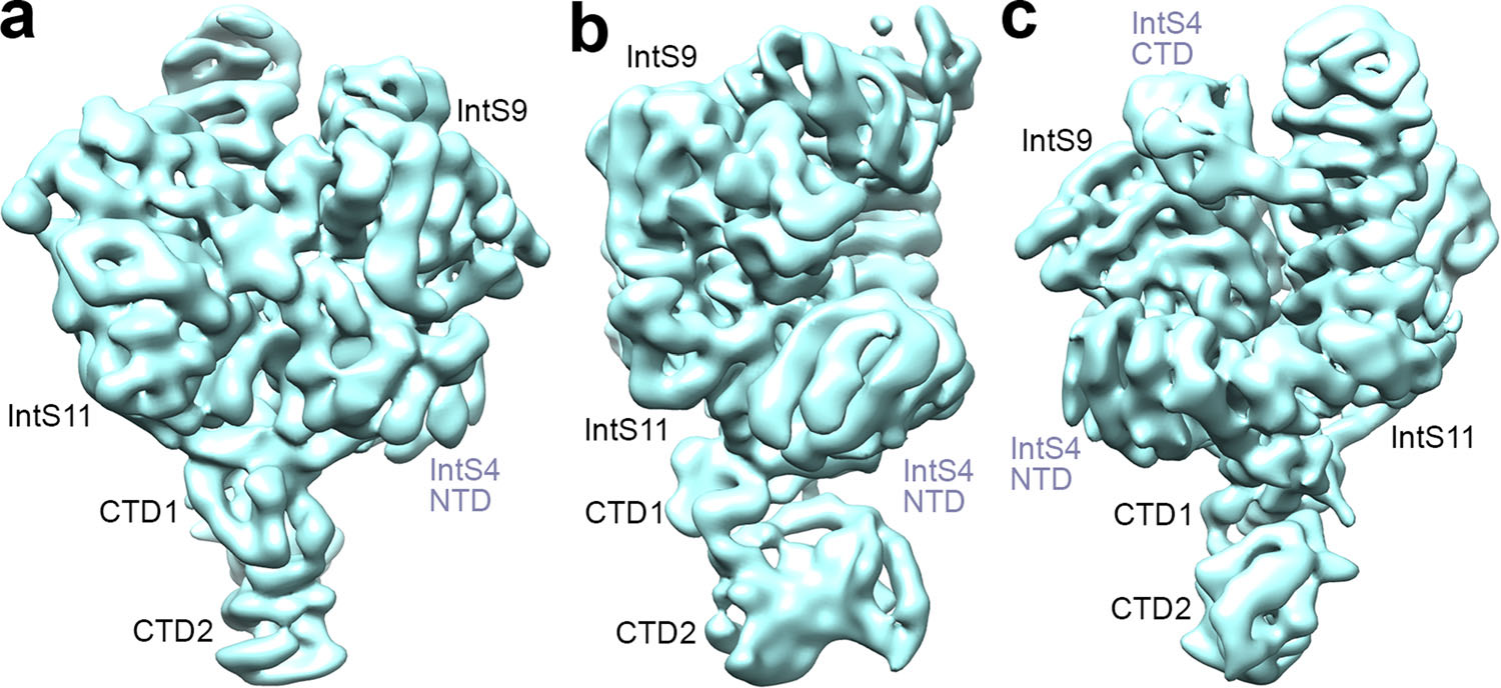
Weak EM density for some regions of the *Drosophila* IntS4-IntS9-IntS11 complex (ICM). **a** Cryo-EM density of the IntS4-IntS9-IntS11 complex low-pass filtered to 7 Å resolution, and viewed after 90° (**b**) and 180° (**c**) rotation around the vertical axis. Weak EM density for CTD2 of IntS9 and IntS11 and residues beyond 570 of IntS4 (CTD) becomes more visible at this resolution, but the quality of the density was not sufficient to allow an atomic model to be built for IntS4 CTD. Panels **a**–**c** created with Chimera^46^.

**Fig. 3:**
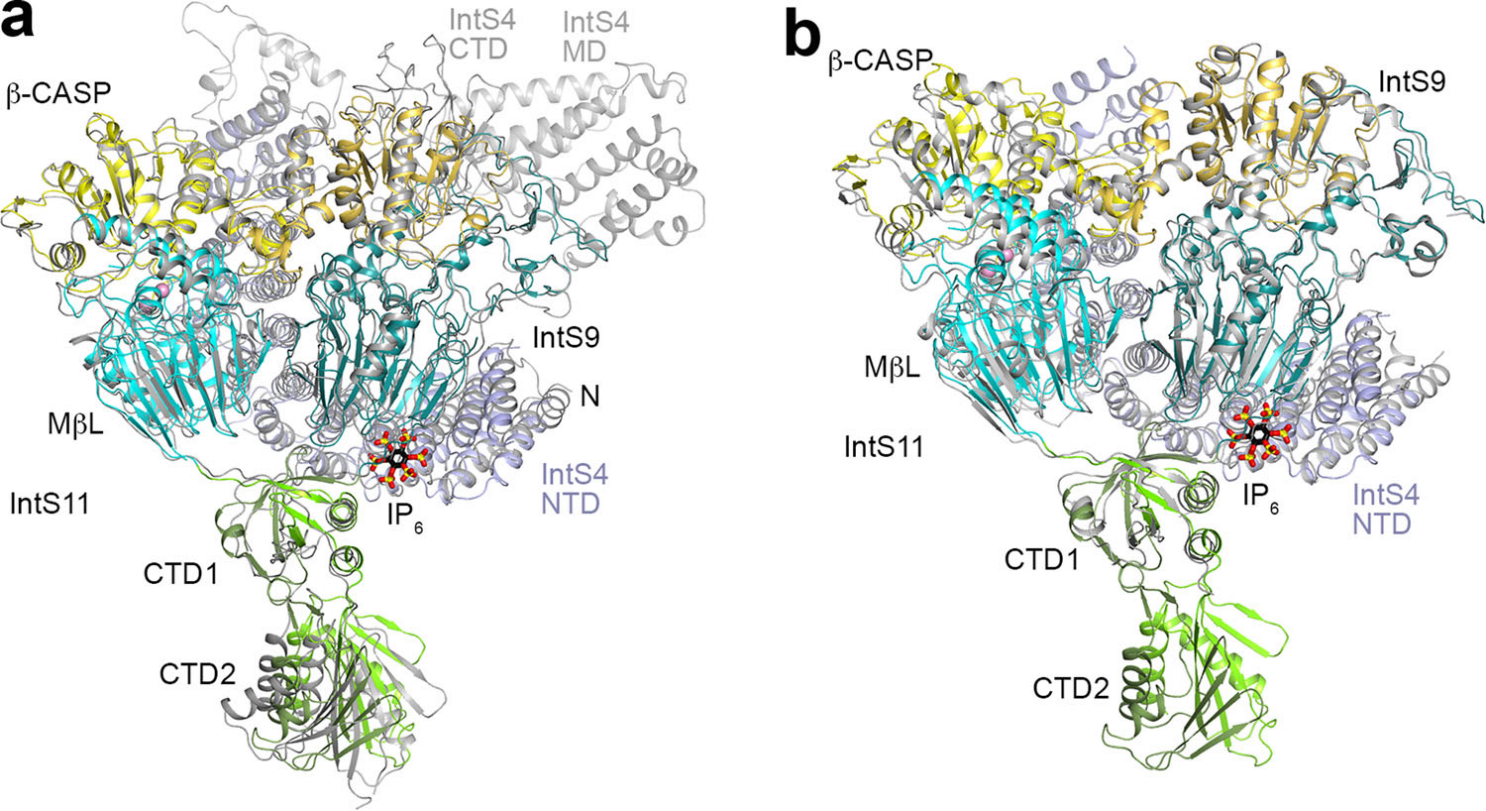
Structural comparison between *Drosophila* and human ICM. **a** Overlay of the overall structure of *Drosophila* ICM and human ICM in the structure of Integrator in complex with PP2A (PDB entry 7CUN). The positions of CTD2 of IntS9 and IntS11 are noticeably different, likely related to the flexibility in these domains. There are differences in the registering of residues in places, and many of the residues in human Integrator lack models for the side chain due to poor EM density. **b** Overlay of the overall structure of *Drosophila* ICM and human ICM (PDB entry 7BFP). The rms distances for equivalent Cα atoms of IntS11, IntS9, and IntS4 are 1.5, 1.3, and 1.6 Å, respectively. With IntS11 in overlay, the orientations of the IntS9 and IntS4 subunits in the two structures differ by 2.6 and 2.9° rotation. Two additional pairs of helices at the C-terminal end of IntS4 NTD are modeled in the *Drosophila* ICM (residues 500–570). Many loops on the surface of the two structures have different conformations (for example residues 300–317 of *Drosophila* IntS11), and some of the loops are missing in human ICM (such as residues 343–370 in IntS9). Conformational differences are also seen for the CTD1 complex, and the CTD2 of IntS9 and IntS11 are not modeled in the human ICM. Differences in registering are also observed, for example see Fig. 5c.

To gain structural insight into the inner workings of IntS11, we co-expressed and purified the *Drosophila* ICM using baculovirus-infected insect cells (Fig. 1a, b) and determined its structure at 2.74 Å resolution by cryo-EM (Figs. 1a, c, d, 2a–c, Table 1, Supplementary Fig. 1). The overall structure of *Drosophila* ICM (Fig. 1c, d) is generally similar to that of human ICM^19,21^ (Fig. 3a, b). IntS11 is in a closed, inactive state in our structure of *Drosophila* ICM, as well as in those reported recently of human ICM^19,21^.

**Table 1:** Cryo-EM data collection, structure refinement, and validation statistics

	*Drosophila* IntS4-IntS9-IntS11 complex (ICM)
**Data collection and processing**	
Magnification	81,000
Voltage (kV)	300
Electron exposure (e^–^/Å^2^)	51
Defocus range (μm)	−1 to −2.5
Pixel size (Å)	1.083
Symmetry imposed	C1
Image stacks (no.)	3083
Initial particles images (no.)	2,920,144
Final particle images (no.)	620,438
Map resolution (Å)	2.74
FSC threshold	0.143
Map sharpening B-factor (Å^2^)	−81.8
**Refinement**	
Number of protein residues	1621
Number of metal ions	2
Number of atoms	12,819
R.m.s. deviations	
Bond lengths (Å)	0.010
Bond angles (°)	1.093
PDB validation	
Clash score	6.34
Poor rotamers (%)	0.07
Ramachandran plot	
Favored (%)	90.11
Allowed (%)	9.89
Disallowed (%)	0.00

The structure shows that the C-terminal segments of IntS9 and IntS11 contain two separate domains, CTD1 and CTD2 (Fig. 1a, c), similar to their paralogs CPSF100 and CPSF73^17^. The two CTD2 domains have weak EM density (Fig. 2a–c, Supplementary Fig. 1), and their atomic models were guided by the structure of the human IntS9-IntS11 CTD2 complex^23^. The CTDs have extensive interactions with each other, facilitating the association of IntS9 and IntS11.

The metallo-β-lactamase and β-CASP domains of IntS9 and IntS11 form a pseudo-dimer in this structure (Fig. 1c), remarkably similar to the pseudo-dimer for the equivalent domains of CPSF100 and CPSF73 in the active U7 machinery (Fig. 1e)^17^. The N-terminal domain (NTD) of IntS4 contacts the metallo-β-lactamase domain of IntS9 and the back face of IntS11 metallo-β-lactamase and β-CASP domains (Fig. 1c, d), which may promote the formation of this pseudo-dimer.

### An unexpected IP_6_-binding site in *Drosophila* ICM

**Fig. 4:**
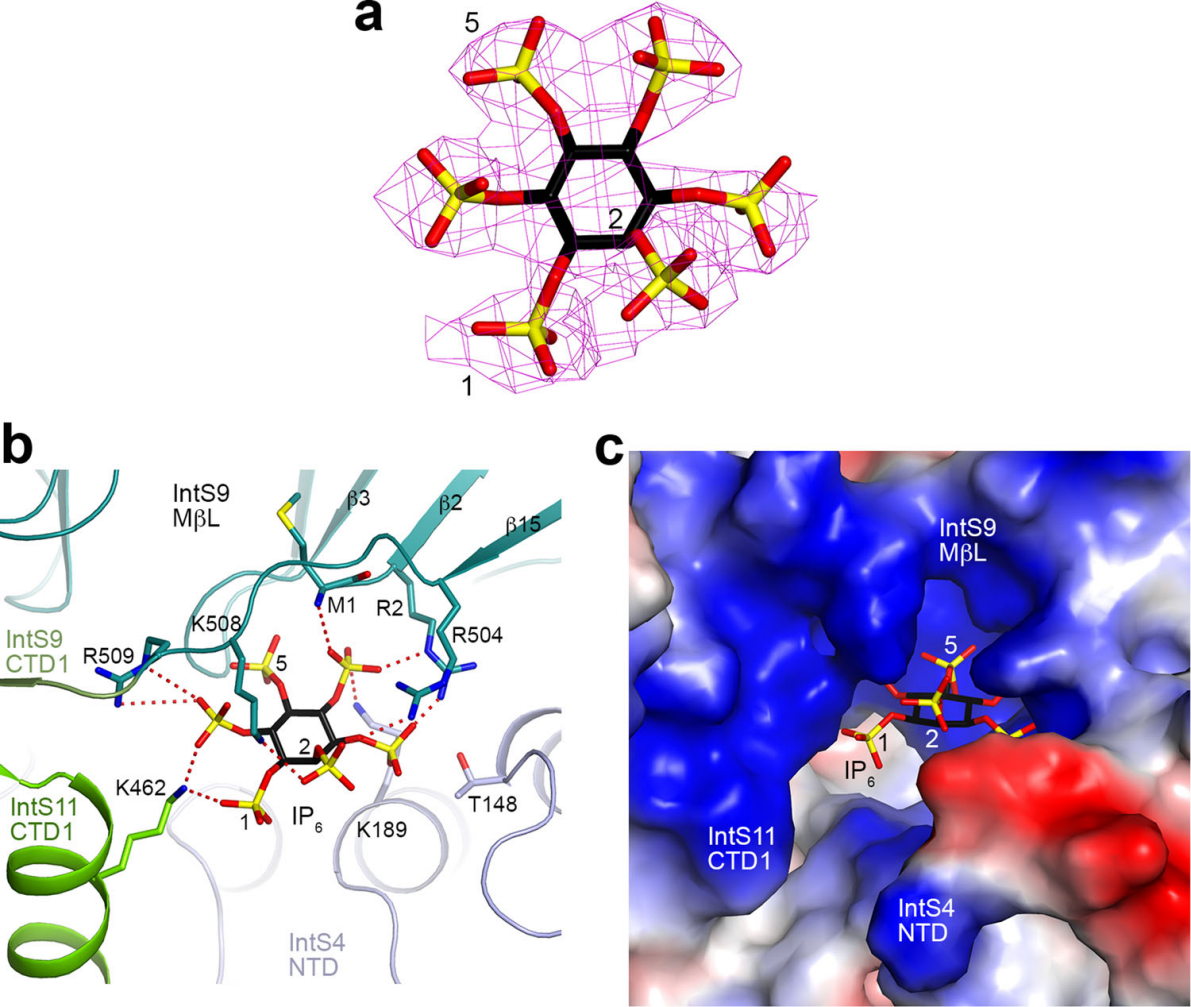
Detailed interactions between IP_6_ and the *Drosophila* IntS4-IntS9-IntS11 complex (ICM). **a** Cryo-EM density for IP_6_. The axial, 2-position is labeled. **b** Detailed interactions between IP_6_ and the ICM. Ionic interactions between the phosphates of IP_6_ and ICM are indicated in dashed lines (red). **c** Electrostatic surface of ICM near the binding site for IP_6_.

The structure of *Drosophila* ICM unexpectedly revealed the presence of an inositol hexakisphosphate (IP_6_) molecule (Fig. 1c, d), with good quality EM density (Fig. 4a). The compound was bound to the ICM during expression in insect cells and remained associated through two column purification steps, suggesting that it has a high affinity for ICM.

IP_6_ is located at an interface among all three subunits of the ICM, formed by the N-terminus and the linker to CTD1 of IntS9, the CTD1 of IntS11, and the first few helical repeats of IntS4 NTD (Fig. 4b). IP_6_ has ionic interactions with all three subunits, including Lys189 of IntS4, the N-terminal ammonium ion, Arg2, Arg504, Lys508 and Arg509 of IntS9, and Lys462 of IntS11 (Fig. 4b). These residues create a large, highly positively charged pocket (Fig. 4c), accommodating IP_6_ or other highly negatively charged molecules.

The EM density for IP_6_ is of sufficient quality such that we can recognize the 2-position of inositol (Fig. 4a). The phosphate at this position is in the axial position, whereas those at the other positions are equatorial. The 2-phosphate interacts with Arg504 and Lys508 of IntS9 (Fig. 4b), suggesting that IP_5_, which lacks this phosphate, would have weaker interactions with ICM. This is consistent with the observation that the EM density for all six phosphates is roughly equivalent (Fig. 4a), suggesting that they are present with similar occupancies. The structure suggests that a pyrophosphate group at the 1 position (1-IP_7_) could be accommodated, while accommodation of such a group at the 5 position (5-IP_7_) could be more difficult (Fig. 4c). Nonetheless, there is little indication of extra EM density at the 1 or the 5 position (Fig. 4a). Further studies are needed to ascertain whether inositol pyrophosphates play a role in Integrator function.

### IP_6_ binding is also found in human ICM

**Fig. 5:**
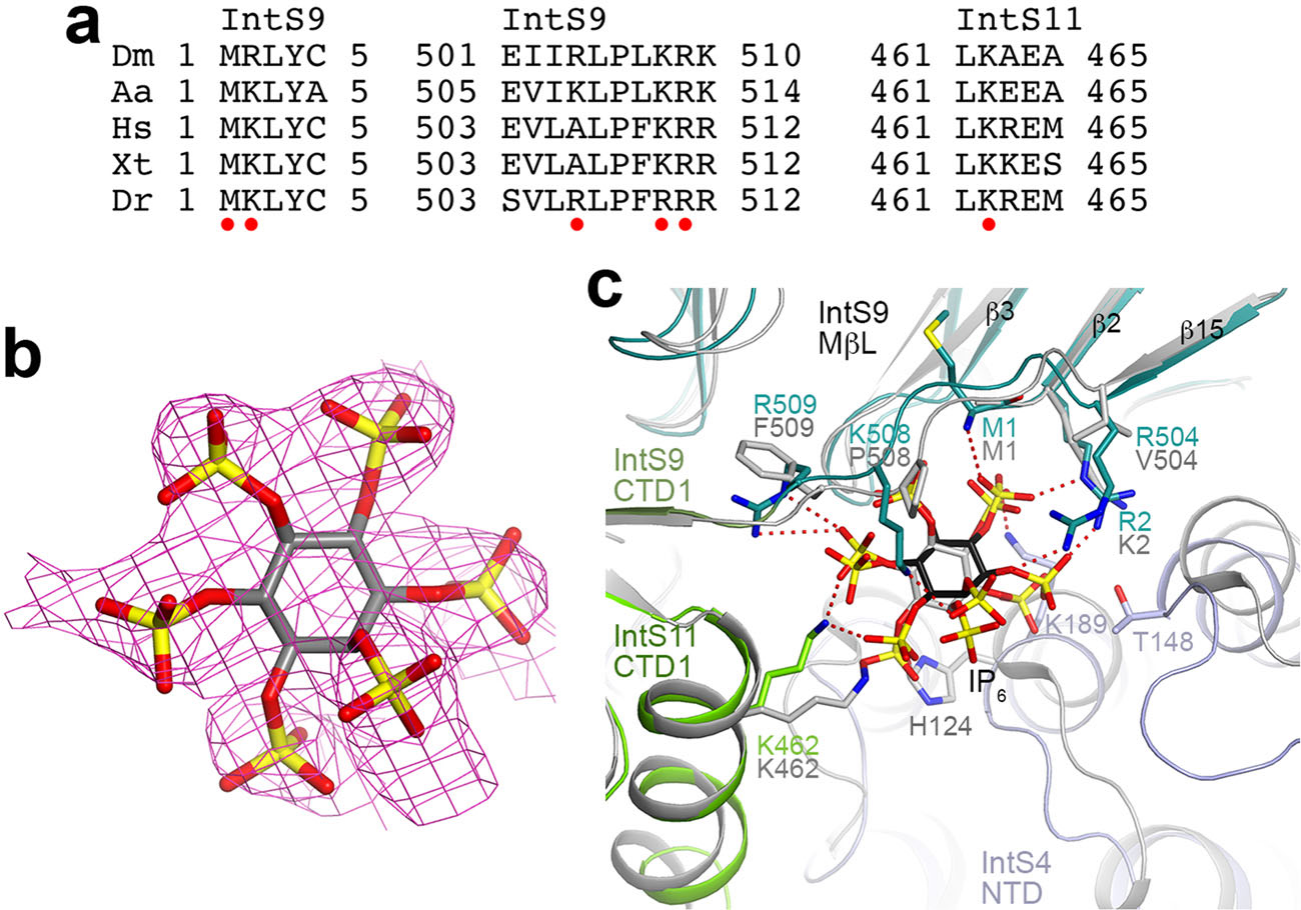
An IP_6_-binding site in human INTS4-INTS9-INTS11 complex (ICM). **a** Alignment of selected sequences for IntS9 and IntS11 residues in the IP_6_ binding site. Residues that have ionic interactions with the phosphate groups on IP_6_ are indicated with the red dots. Dm *D. melanogaster*, Aa *Aedes aegypti*, Hs *H. sapiens,* Xt *Xenopus tropicalis*, Dr *Danio rerio*. **b** Cryo-EM density for IP_6_ in human ICM (EMDB 12159)^19^. An IP_6_ molecule was built into the density and subjected to real-space refinement. **c** Overlay of the structures of *Drosophila* ICM (in color) and human ICM (gray, PDB entry code 7BFP) near the IP_6_ binding site. The human ICM structure likely has a registering shift of two residues for residues 504–509 of INTS9. Arg504, Lys508, and Arg509 of *Drosophila* IntS9 would be equivalent to Ala506, Lys510, and Arg511 instead of Val504, Pro508, and Phe509 of human INTS9, which would also be consistent with the sequence conservation in this region (**a**).

Residues in the IP_6_ binding site are generally conserved among IntS9 and IntS11 homologs (Fig. 5a). While Lys189 is glutamine in vertebrate IntS4, IP_6_ should maintain favorable interactions with the dipoles of the helices in the IntS4 NTD.

In the structure of the human ICM^19^, EM density highly consistent with IP_6_ is present near the N-terminus of INTS9 as well (Fig. 5b), although IP_6_ was not included in that atomic model. The detailed binding mode of IP_6_ in human ICM has only slight differences compared to that in *Drosophila* ICM (Fig. 5c). In the structure of the human Integrator-PP2A complex (EMDB 30473)^21^, some EM density is present near the N-terminus of INTS9, although the quality of the density here is poor. In fact, a few N-terminal residues of INTS9 were built into this density. Overall, the structural observations suggest that this pocket is likely to bind IP_6_ or another negatively charged compound(s) and play a role in the function of ICM in general.

### Mutations in IP_6_-binding site block IP_6_ binding

**Fig. 6:**
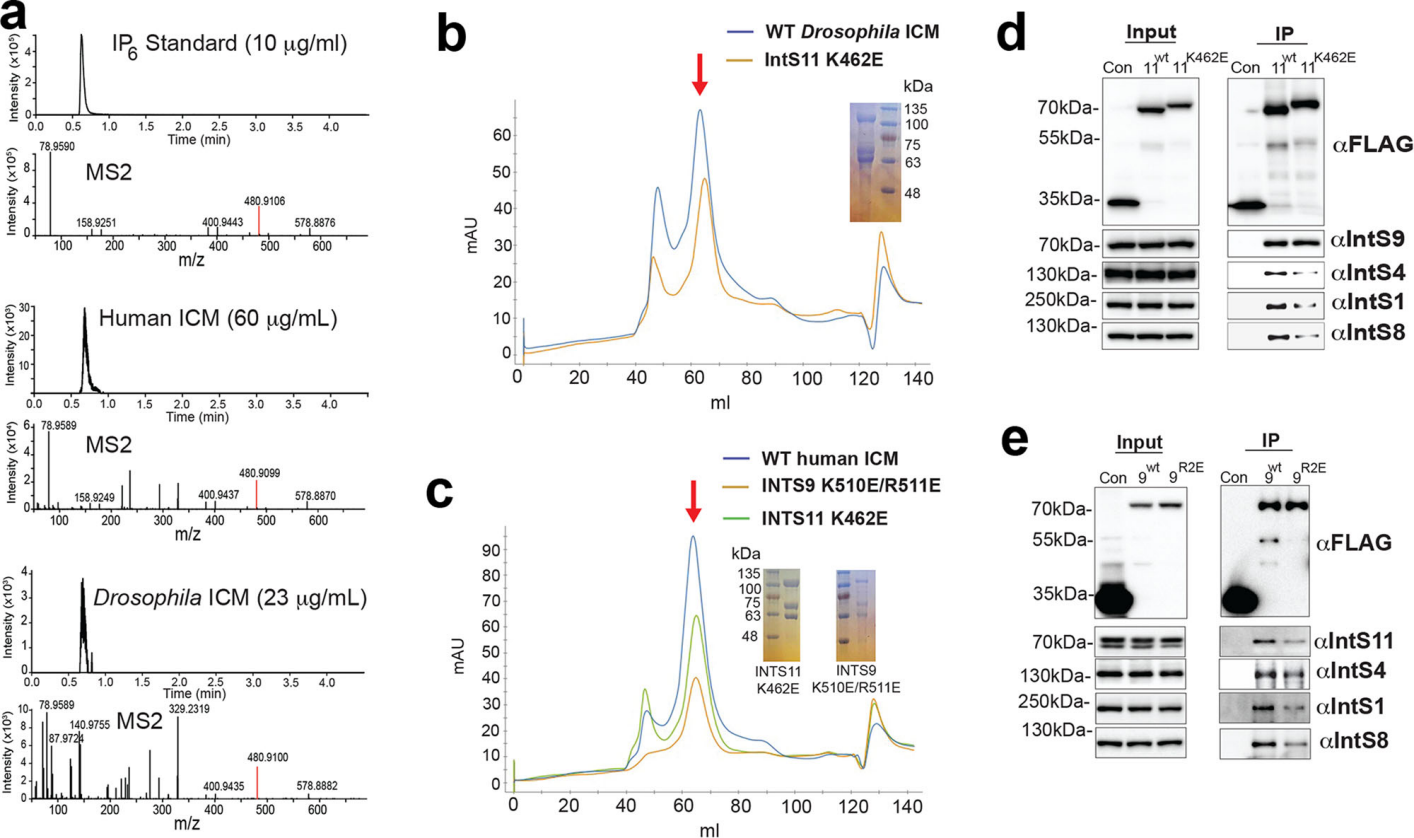
Mutations in the IP_6_-binding site do not completely abolish Integrator assembly. **a** Chromatograms (upper panel) and fragmentation spectra (lower panel) for the IP_6_ [M-2H] ion for IP_6_ standard, wild-type human ICM, and wild-type *Drosophila* ICM. The IP_6_ signal for *Drosophila* ICM is weaker due to the lower protein concentration. **b** Gel filtration profiles of wild-type (WT) and K462E mutant of *Drosophila* ICM, and SDS gel of the mutant. The position of the complex is indicated with the red arrow. The experiment for the mutant was done one time. mAU milli Absorption Unit. **c** Gel filtration profiles of wild-type, K462E, and K510E/R511E mutants of human ICM, and SDS gels of the mutants. The experiment for the mutants was done one time. **d** Western blot analysis of input nuclear extracts (left) and IP (right) from DL1 cells stably expressing FLAG-tagged proteins as indicated. IP conducted using anti-FLAG affinity resin was normalized to FLAG signals in each IP. The IntS11-K462E mutation reduced the pulldown of IntS1, IntS4, and IntS8, but had no effect on IntS9 pulldown. The western blot shown is representative of two independent experiments that were done. **e** Western blot analysis of input nuclear extracts (left) and IP (right) from DL1 cells stably expressing FLAG-tagged proteins as indicated. The IntS9-R2E mutation reduced the pulldown of IntS1, IntS8, and IntS11, but had only minor effect on IntS4 pulldown. The western blot shown is representative of two independent experiments that were done. Source data are provided as a Source Data file.

To characterize the importance of IP_6_ for Integrator, we first created mutations in the IP_6_ binding site that changed the positively charged residues to negatively charged ones and assessed their impact on IP_6_ binding and Integrator assembly. We developed a protocol that enabled us to confirm by mass spectrometry the binding of IP_6_ to wild-type human or *Drosophila* ICM samples purified from insect cell expression (Fig. 6a). We then produced both *Drosophila* and human ICM samples containing the K462E mutation in IntS11 and the human ICM sample containing the K510E/R511E double mutation in IntS9. The residue equivalent to Arg504 of *Drosophila* IntS9 is Ala in human IntS9 (Fig. 5a), and hence it was not mutated. The mutants produced gel filtration profiles that are comparable to those of wild-type ICMs (Fig. 6b, c), but we failed to detect IP_6_ in our mass spectrometry experiments on them (Supplementary Fig. 2).

We also created *Drosophila* DL1 nuclear extracts from cell lines stably expressing FLAG-IntS11-WT, FLAG-IntS11-K462E, FLAG-IntS9-WT, or FLAG-IntS9-R2E and then purified associated complexes using anti-FLAG affinity. We observed that the IntS11-K462E and the IntS9-R2E mutations reduced but did not completely abolish association with Integrator subunits (Fig. 6d, e). These data confirm the existence of the IP_6_ binding site in *Drosophila* and human Integrator and show that mutations of residues in this binding site can block IP_6_ binding but do not completely abolish Integrator assembly.

### IP_6_ is required for Integrator function

**Fig. 7:**
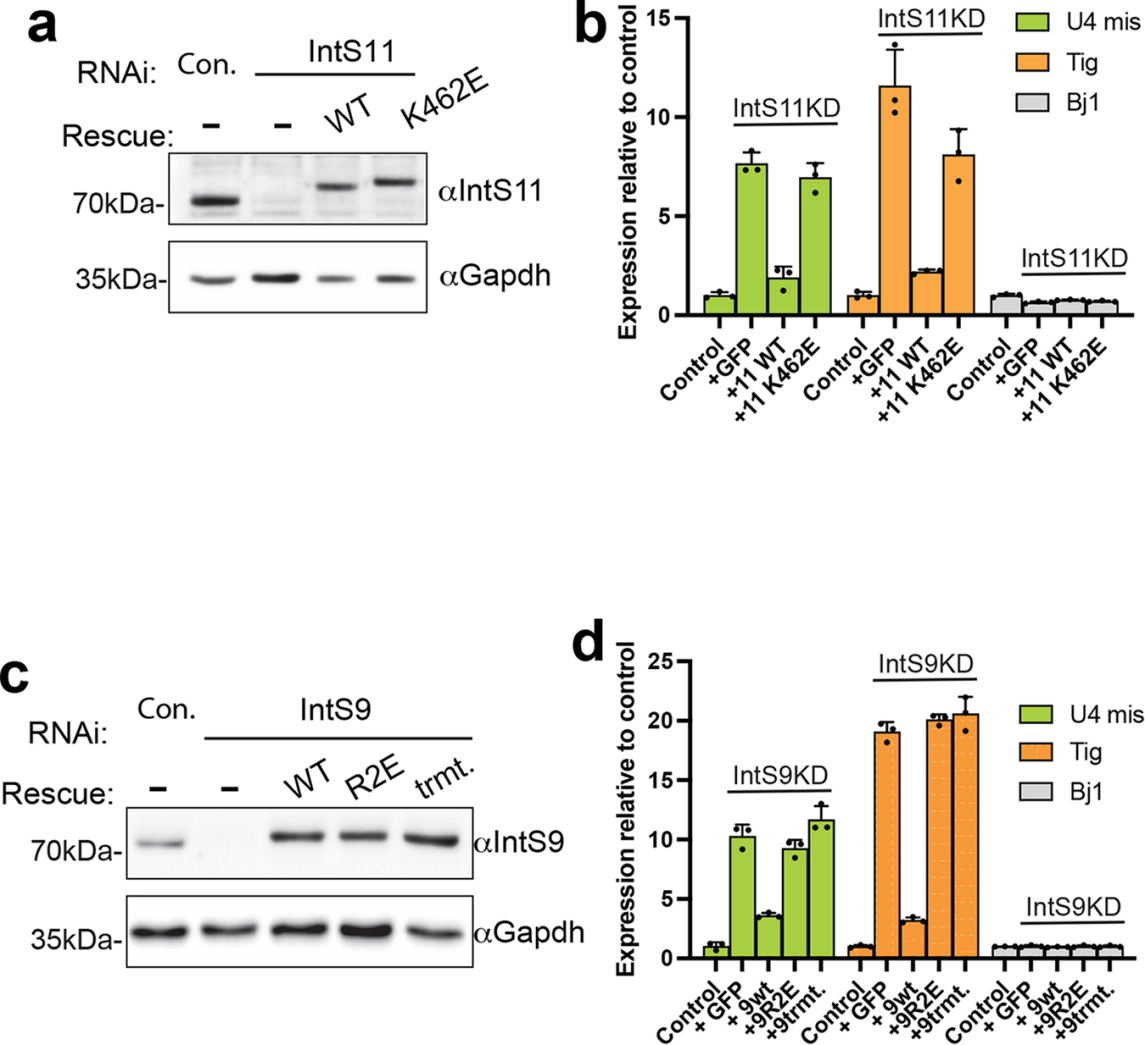
IP_6_ is important for Integrator function. **a** Western blot analysis of DL1 cell lysates derived from cells treated with either control or IntS11 dsRNA. Rescues were conducted in cells depleted of IntS11 through the inducible expression of RNAi-resistant cDNA. **b** RT-qPCR analysis of selected genes identified to be upregulated upon IntS11 depletion using RNA-seq. Results are shown from biologically independent replicates, depicting averages and standard deviations (mean ± SD, *n* = 3). **c** Western blot analysis of DL1 cell lysates derived from cells treated with either control or IntS9 dsRNA. Rescues were conducted in cells depleted of IntS9 through the inducible expression of RNAi-resistant cDNA. trmt *Drosophila* IntS9 R504E/K508E/R209E triple mutant. **d** RT-qPCR analysis of selected genes identified to be upregulated upon IntS11 depletion using RNA-seq. Results are shown from biologically independent replicates, depicting averages and standard deviations (mean ± SD, *n* = 3). Source data are provided as a Source Data file.

To determine the functional significance of IP_6_ binding to Integrator, we devised a method to induce expression of IntS11-WT or IntS11-K462E in cells where endogenous IntS11 has been depleted using dsRNA targeting its 5′ and 3′ UTRs. We observed that treatment of DL1 cells with IntS11 dsRNA resulted in effective depletion of endogenous IntS11, whereas induction of IntS11 transgenes allowed expression of near endogenous levels of IntS11-WT or IntS11-K462E proteins (Fig. 7a). We then analyzed the impact of IntS11 depletion on U4snRNA processing, transcriptional attenuation of a previously validated mRNA-encoding gene called tigrin (tig)^6^, and a gene found not to be regulated by Integrator (Bj1). As expected, depletion of IntS11 resulted in a significant increase in U4snRNA misprocessing as well as tig transcription but did not affect Bj1 (Fig. 7b). Importantly, these phenotypes were restored upon re-expression of IntS11-WT but could not be rescued by the IntS11-K462E mutant (Fig. 7b). Similar observations were made with the R2E and the R504E/K508E/R209E triple mutation (trmt) in IntS9 (Fig. 7c, d). These results show that although these mutations do not completely abolish the assembly of Integrator subunits, they are significantly disruptive to Integrator function, underscoring the critical need for IP_6_ binding for Integrator activity.

**Fig. 8:**
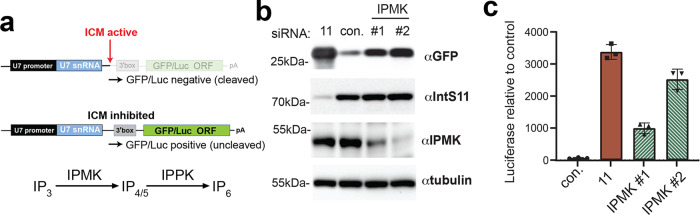
Downregulation of IP_6_ biosynthesis disrupts Integrator function. **a** Schematic of the U7snRNA reporter systems, where the U7 gene is cloned upstream of GFP or luciferase. Top: in WT cells, the ICM drives RNA Pol II termination and prevents GFP/luc expression. Bottom: Upon losing ICM function through IPMK depletion, RNA Pol II productively elongates through the GFP/luc ORF, yielding expression. The Integrator cleavage site is indicated with the red arrow. **b** Western blot analysis of 293 T cell lysates treated with siRNA as labeled and the U7-GFP reporter. The levels of IntS11 and IPMK depletion are shown, and the level of GFP expression from the reporter. **c** Similar experiment as in panel **b** except the U7-luc reporter was used, and light units were measured in each case. Results are shown from biologically independent replicates, depicting averages and standard deviations (mean ± SD, *n* = 3). Source data are provided as a Source Data file.

Finally, we transfected cells with two different siRNAs targeting inositol polyphosphate multikinase (IPMK), an upstream kinase required for both IP_5_ and IP_6_ biosynthesis^28,29^. We assessed Integrator function using two reporters where the U7snRNA promoter, gene body, and 3′ cleavage site are placed upstream of either GFP or luciferase with the rationale that loss of Integrator function leads to transcriptional readthrough and expression of the downstream open reading frames (Fig. 8a). We observed that depletion of IPMK resulted in increased expression of GFP and luciferase relative to control (Fig. 8b, c). Notably, the siRNA capable of more significant IPMK depletion (#2 in Fig. 8b, c) produced much higher GFP/luciferase expression, in fact reaching a level similar to that observed after depletion of IntS11. These data further reveal the critical nature of IP_6_ in Integrator function and Pol II transcription.

The binding site for IP_6_ in ICM is located far (55 Å) from the active site of IntS11 and is on the opposite face from the opening for the canyon in IntS11 for binding RNA (Fig. 1c). Therefore, this binding site is unlikely to affect the catalysis by IntS11 directly. In addition, the IP_6_ binding site is far away from other modules of Integrator and Pol II in the reported structures (Supplementary Figs. 3a, b)^21,26,27^, and therefore IP_6_ likely exerts its effects on Integrator solely through the ICM. Mutation of Lys462 also affected human Integrator function, and it was suggested this binding site could interact with a part of the snRNA substrate^19^, although our functional studies have demonstrated a critical role for IP_6_ in Integrator activity. The binding of IP_6_ is also observed in structures of the spliceosome^30,31^, although the function of this binding has not been reported. Our observations expand the repertoire of IP_6_ as a molecule impacting RNA editing and processing^32–34^ and other processes in the cell^35,36^.

## Methods

### Protein expression and purification

*Drosophila* IntS4, IntS9, and IntS11 were co-expressed in insect cells. IntS9 and IntS11 were cloned into the pFL acceptor vector. N-terminal 6xHis-tagged IntS4 was cloned into the pSPL donor vector. These two vectors were fused by Cre recombinase. Tni insect cells (Expression Systems) (2 × 10^6^ cells·ml^–1^) were infected with 16 ml of IntS4-IntS9-IntS11 P2 virus and harvested after 48 h.

For purification, the cell pellet was resuspended and lysed by sonication in 100 ml of buffer containing 20 mM Tris (pH 8.0), 250 mM NaCl, 2 mM βME, 5% (v/v) glycerol, and one tablet of protease inhibitor mixture (Sigma). The cell lysate was then centrifuged at 15,000 × *g* for 40 min at 4 °C. The protein complex was purified from the supernatant via nickel affinity chromatography (Qiagen). The protein complex was further purified using a Hiload 16/60 Superdex 200 column (Cytiva). The IntS4-IntS9-IntS11 complex was concentrated to 2 mg·ml^–1^ in a buffer containing 20 mM Tris (pH 8.0), 300 mM NaCl, and 2 mM DTT, and stored at −80 °C.

### EM specimen preparation and data collection

All specimens for cryo-EM were frozen with an EM GP2 plunge freezer (Leica) set at 20 °C and 99% humidity. Cryo-EM imaging was performed in the Simons Electron Microscopy Center at the New York Structural Biology Center using Leginon^37^.

For the IntS4-IntS9-IntS11 complex, a 3.5 μL aliquot at 0.1 mg·ml^–1^ was applied to one side of a Quantifoil 400 mesh 1.2/1.3 gold grid with graphene oxide support film (Quantifoil). After 30 s, the grid was blotted for 1.5 s on the other side and plunged into liquid ethane. 3083 image stacks were collected on a Titan Krios electron microscope at New York Structural Biology Center, equipped with a K3 direct electron detector (Gatan) at 300 kV with a total dose of 51 e^−^ Å^−2^ subdivided into 40 frames in 2 s exposure using Leginon. The images were recorded at a nominal magnification of 81,000× and a calibrated pixel size of 1.083 Å, with a defocus range from –1 to −2.5 μm.

### Image processing

Image stacks were motion-corrected and dose-weighted using RELION 3.1^38^. The patch CTF parameters were determined with cryoSPARC^39^. First, 2,920,144 particles were auto-picked and subjected to 2D classification in cryoSPARC. 1,149,821 particles in classes with recognizable features by visual inspection were used to generate eight 3D initial models by ab initio reconstruction. After one round of heterogeneous refinement, 620,438 particles were imported to RELION for CTF refinement and Bayesian polishing, yielding a map at 2.74 Å resolution. An analysis of the map gave sphericity of 0.963^40^, suggesting that anisotropy is not a problem for this reconstruction.

### Model building

Atomic models for IntS11, IntS9, and IntS4 were built manually into the cryo-EM density with Coot^41^. Homology models for *Drosophila* IntS9 and IntS11 were generated with I-TASSER^42^, based on the structures of human CPSF100 and CPSF73^43^. The atomic models were improved by real-space refinement with the program PHENIX^44^.

The model of IP_6_ in the *Drosophila* ICM was placed in the human ICM cryo-EM density (EMDB 12159)^19^ and manually adjusted to fit the density. Real-space refinement was then used to optimize the fitting of IP_6_ to the EM density.

### LC-MS/MS analysis of IP_6_ binding

Samples were diluted 100x in 20% (v/v) acetonitrile to help denature the complex and reduce the salt concentration, after which they were placed into clean autosampler tubes and analyzed via LC-MS/MS.

IP_6_ analysis was carried out on a Dionex Ultimate 3000 UHPLC coupled to a Q Exactive Plus mass spectrometer (Thermo Fisher). IP_6_ was separated on an Accucore aQ C18 2.1 ×150 mm column (Thermo Fisher). The mobile phases were A: 20 mM ammonium acetate, pH 9.0, and B: acetonitrile. The flow rate was set to 400 μL/min, and the column oven was set to 25 °C. 40 μL of each sample was injected, and the analytes were eluted isocratically at 20% B. The gradient was then ramped to 50% B to wash the column and returned to starting conditions for re-equilibration. The total runtime was 4.5 min.

The Q Exactive Plus was operated in negative mode with a heated electrospray ionization (HESI) source, in conjunction with a parallel reaction monitoring (PRM) method. An IP_6_ standard was used to determine the [M-H] mass (658.8543) and [M-2H] mass (328.9238), both of which were isolated and fragmented to increase the confidence of IP_6_ identification. The standard was also used to determine fragment ions, and retention times, and to optimize the collision energy. Precursor ions were isolated with a 2.0 *m/z* isolation width and then fragmented in the collision cell with a collision energy of 30. The maximum injection time was set to 100 ms, while the AGC target was set to 2e5. The resulting fragment ions were detected in the Orbitrap with a resolution of 17,500 at m/z 200. Fragment ions 560.8766 m/z [M − H] and 480.9104 *m/z* [M − 2H] were used to identify the analytes. Chromatograms were extracted with a 10 ppm mass error, and peaks were integrated using XCalibur software (Thermo Fisher).

### Plasmid construction and stable cell lines generation

For mutation analysis of *Drosophila* IntS9 and IntS11, site-directed PCR mutagenesis was used to create the IntS9-R2E, IntS9-trmt, and IntS11-K462E mutations (primer sequences are in Supplementary Table 1). Wild-types and the mutants of dIntS9 and IntS11 were subsequently cloned into the pMT-3xFLAG-puro vector^6^ to express in DL1 cells inducibly. All plasmids were sequenced to confirm identity. To generate cells stably expressing the FLAG-IntS9-WT, FLAG-IntS9-R2E, FLAG-IntS9-trmt, FLAG-IntS11-WT, FLAG-IntS11-K462E, and eGFP control transgenes, 2×10^6^ cells were first plated in regular maintenance media in a 6-well dish overnight. Two micrograms of expressing plasmids were transfected using Fugene HD (Promega, #E2311). After 24 h, 2.5 μg/mL puromycin was added to the media to select and maintain the cell population.

### Nuclear extract preparation

Five 150 mm dishes of each condition of confluent cells (pretreated with 500 μM CuSO_4_ for 24 h) were collected and washed in cold PBS before being resuspended in ten times volumes of the cell pellet of Buffer A (10 mM Tris pH 8, 1.5 mM MgCl_2_, 10 mM KCl, 0.5 mM DTT, and 0.2 mM PMSF). Resuspended cells were allowed to swell during a 15 min rotation at 4 °C. After pelleting down at 1000 × *g* for 10 min, two times volumes of the original cell pellet of Buffer A were added, and cells were homogenized with a dounce pestle B for 40 strokes on ice. Nuclear and cytosolic fractions were separated by centrifuging at 800 × *g* for 10 min. To attain a nuclear fraction, the pellet was washed once with Buffer A before being resuspended in two times volumes of the original cell pellet of Buffer C (20 mM Tris pH 8, 420 mM NaCl, 1.5 mM MgCl_2_, 25% (v/v) glycerol, 0.2 mM EDTA, 0.5 mM PMSF, and 0.5 mM DTT). The samples were then homogenized with a dounce pestle B for 20 strokes on ice and rotated for 30 min at 4 °C before centrifuging at 15,000 × *g* for 30 min at 4 °C. Finally, supernatants were collected and subjected to dialysis in Buffer D (20 mM HEPES, 100 mM KCl, 0.2 mM EDTA, 0.5 mM DTT, and 20% (v/v) glycerol) overnight at 4 °C against a 3.5 kDa MWCO membrane (Spectrum Laboratories, #132720). Prior to any downstream applications, nuclear extracts were centrifuged again at 15,000 × *g* for 3 min at 4 °C to remove any precipitate.

### Western blotting and anti-FLAG affinity purification

To check protein expression, cells were lysed directly in wells in 2× SDS sample buffer (120 mM Tris pH 6.8, 4% SDS, 200 mM DTT, 20% (v/v) glycerol, and 0.02% bromophenol blue). Lysates were incubated at room temperature with periodic swirling before a 10 min boiling at 95 °C and a short sonication. Denatured protein samples were then resolved in a 10% SDS-PAGE and transferred to a PVDF membrane (Bio-Rad, #1620177). Blots were probed by custom-designed *Drosophila* antibodies as previously described^6^ diluted in PBS-0.1% Tween supplemented with 5% nonfat milk. To detect proteins from 293 T lysate, anti-hInts11 (Bethyl, #A301-274A), anti-hIMPK (Thermo, #PA5-21629), anti-GFP (Clontech, #632381), anti-alpha Tubulin (abcam, #ab15246), and anti-GAPDH (Thermo, #MA5-15738) were used at the dilution suggested by the manufacturer.

To purify FLAG-tagged Integrator complexes, 1 mg of nuclear extract was mixed with 40 μL anti-Flag M2 affinity agarose slurry (Sigma, #A2220) equilibrated in binding buffer (20 mM HEPES pH 7.4, 100 mM KCl, 10% (v/v) glycerol, 0.1% NP-40) and rotated for 2 h at 4 °C. Following the 2 h incubation/rotation, five sequential washes were carried out in binding buffer with a 10 min rotation at 4 °C followed by a 500 × *g* centrifugation at 4 °C. After the final wash, the binding buffer supernatant was removed using a pipette, and the protein complexes were eluted from the anti-FLAG resin by adding 40 μL of 2× sample buffer and boiled at 95 °C for 5 min. For input samples, nuclear extracts were mixed with 5× loading buffer and boiled, and 1/10 volume of the immunoprecipitation reaction was loaded on SDS-PAGE.

### RNA Interference

Double-stranded RNAs targeting the 5′ and 3′ UTRs of *Drosophila* IntS11 and an RNAi-resistant region of the IntS9 constructs were generated by in vitro transcription of PCR templates containing the T7 promoter sequence on both ends using MEGAscript kit (Thermo, #AMB13345). For RNA interference experiments, 1.5 × 10^6^/ml of DL1 cells were washed into serum-free media and seeded into a 6-well plate along with 10 μg of dsRNA. After a 1 h incubation, 2 ml of complete growth medium was added, followed by 60 h of incubation before harvest. To perform rescue experiments while knocking down, cells were also treated with 100 μM CuSO_4_ throughout the 60 h incubation period to induce expression of the RNAi-resistant FLAG-IntS9-WT, FLAG-IntS9-R2E, FLAG-IntS9-trmt, FLAG-IntS11-WT, FLAG-IntS11-K462E transgenes.

IntS11 (Sigma, #SASI_Hs01_00032429), IPMK (Sigma, #SASI_Hs01_00047017, top strand sequence CAAACGAUUUAUACCUAAA[dT][dT], and #SASI_Hs01_00047015, GGUUUAUGCUGCUGACUGU[dT][dT]), and control (Sigma, #SIC002) siRNAs (2 μL each of 20 mM stock) were incubated in 50 μL of prewarmed (room temperature) Opti-MEM I reduced serum medium (GIBCO) for 5 min at room temperature. Similarly, RNAiMax (2 μL per well) was incubated in 50 μL prewarmed Opti-MEM I reduced serum medium for 5 min. The siRNA and RNAiMAX dilutions were mixed and incubated for 20 min at room temperature. 2 × 10^5^ 293 T cells were seeded into a 24-well plate, and the prepared transfection mixes of 40 pmols siRNAs were added to each well. Cells were transfected with 60 pmols of siRNA a second time after 24 h of incubation. The cells were expanded into 12-well plate after a total of 48 h and harvested at a total of 72 h of incubation under standard mammalian cell culture conditions.

### Reporter cell lines establishment and luciferase measurement

To construct a reporter plasmid, pAAVS1-TLR targeting vector (Addgene, #64215) was cut with CalI and PspXI to substitute with U11/U7 small nuclear RNA (500 bp upstream of transcription start site, coding region, and 50 bp downstream of coding region) followed by Renilla luciferase/GFP coding region and SV40 poly(A) signal. The reporter plasmid was co-transfected with the gRNA cloned into pU6-(BbsI) CBh-Cas9-T2A-mCherry (Addgene, #64324), targeting AAVS1 locus into 293 T. Briefly, an equal amount of plasmids were transfected with lipofectamine 2000 for 24 h before selecting with 800 ng/ml puromycin for 2 days. Cells were grown in a regular medium without puromycin for a week before clonal selection.

Renilla Luciferase Assay System (Promega, #E2810) was used to assess luciferase activity in the clonal reporter cell lines. The line with the highest luciferase activity after hIntS11 knockdown was selected. To test the role of IPMK in Integrator’s function, the reporter cell lines were seeded in 24-well plate and knocked down twice with siRNAs as described. Luciferase activity was measured according to the manufacturer’s protocol. The background luciferase activity of each sample was calculated by interpolation and subtraction. Briefly, scatterplot and trendline were plotted by protein quantity versus luciferase activity obtained from the same amount of reporter cell line lysate, and an increased amount of the lysate was measured. The bars represent the average of triplicate biological repeats.

### RT-qPCR quantification and analysis

Data were analyzed using the ΔΔCt method with Rps17 as the reference gene and LacZ dsRNA-treated cells as the control, and all PCR amplicon primers were described previously^45^.

### Reporting summary

Further information on research design is available in the Nature Research Reporting Summary linked to this article.

## Supplementary information

Supplementary Information

Reporting Summary

## Data Availability

The structure of the *Drosophila* ICM-IP_6_ complex has been deposited at the PDB under accession code 7SN8. The cryo-EM map of the *Drosophila* ICM-IP_6_ complex has been deposited at the EMDB under accession code 25214. Source data are provided with this paper.

## Source data

Source Data
