# Supplementary material for: Inositol hexakisphosphate is required for Integrator function
Source: Nat Commun. 2022 Sep 30;13:5742. doi: 10.1038/s41467-022-33506-3 (PMC9525679; doi:10.1038/s41467-022-33506-3)
Supplement: Supplementary file 1 — Supplementary Information [file 41467_2022_33506_MOESM1_ESM.pdf]

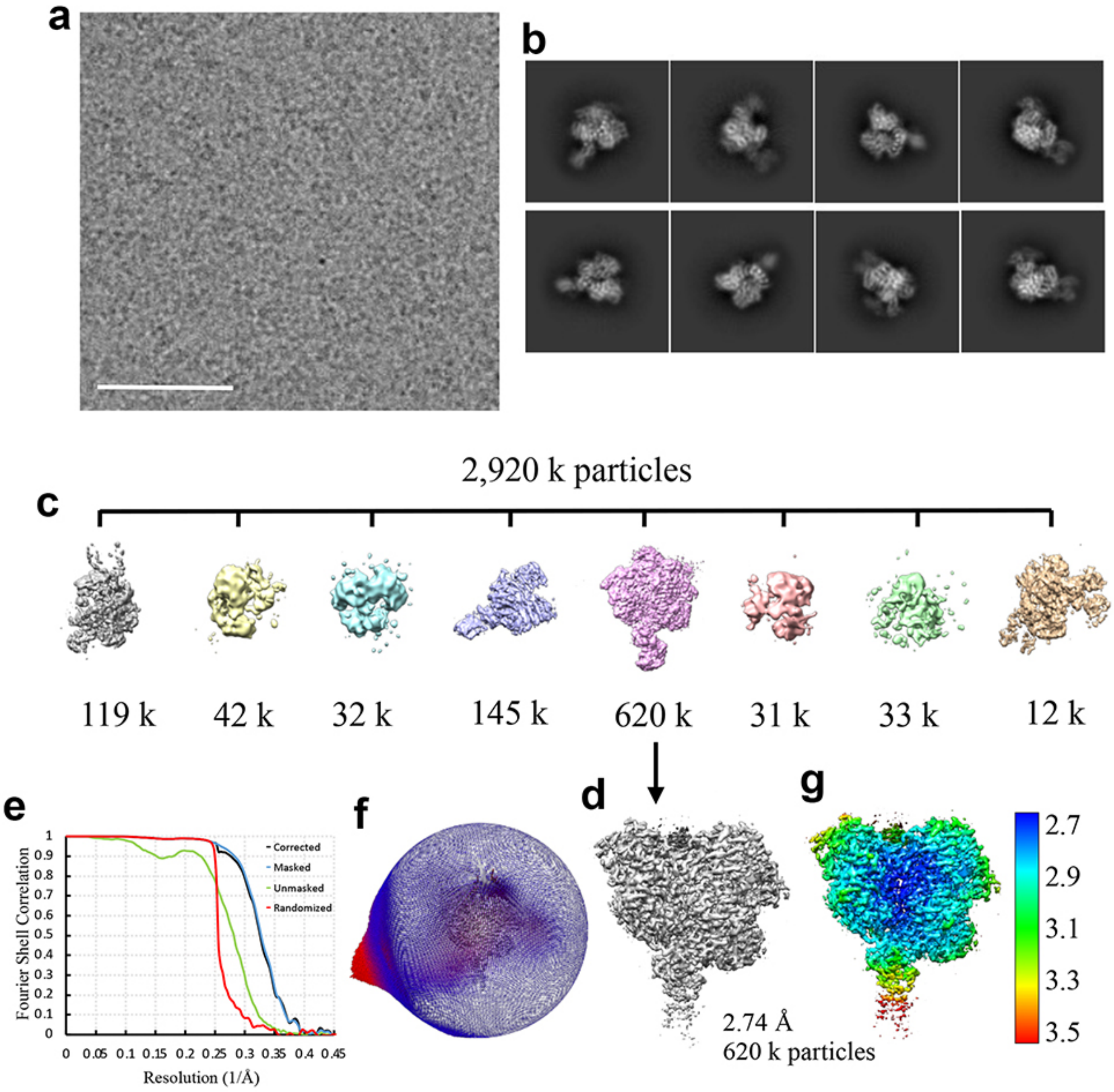

Supplementary Fig. 1. Single-particle cryo-EM analysis of the *Drosophila* IntS4-IntS9-IntS11 complex. (a). A region of a cryo-EM micrograph of the IntS4-IntS9-IntS11 complex. Scale bar: 50 nm. One grid was examined. Most of the image stacks contain particles of similar quality. **(b).** Ten 2D class averages from the cryo-EM micrographs. **(c).** 3D classification (hetero refinement) of particles identified from 2D class averages. **(d).** Final cryo-EM map after Bayesian polishing and refinement in RELION. **(e).** Fourier shell correlation curves for the final model. **(f).** Orientations of particles used in the refinement for the final cryo-EM reconstruction. **(g).** Local resolution map for the final cryo-EM reconstruction.

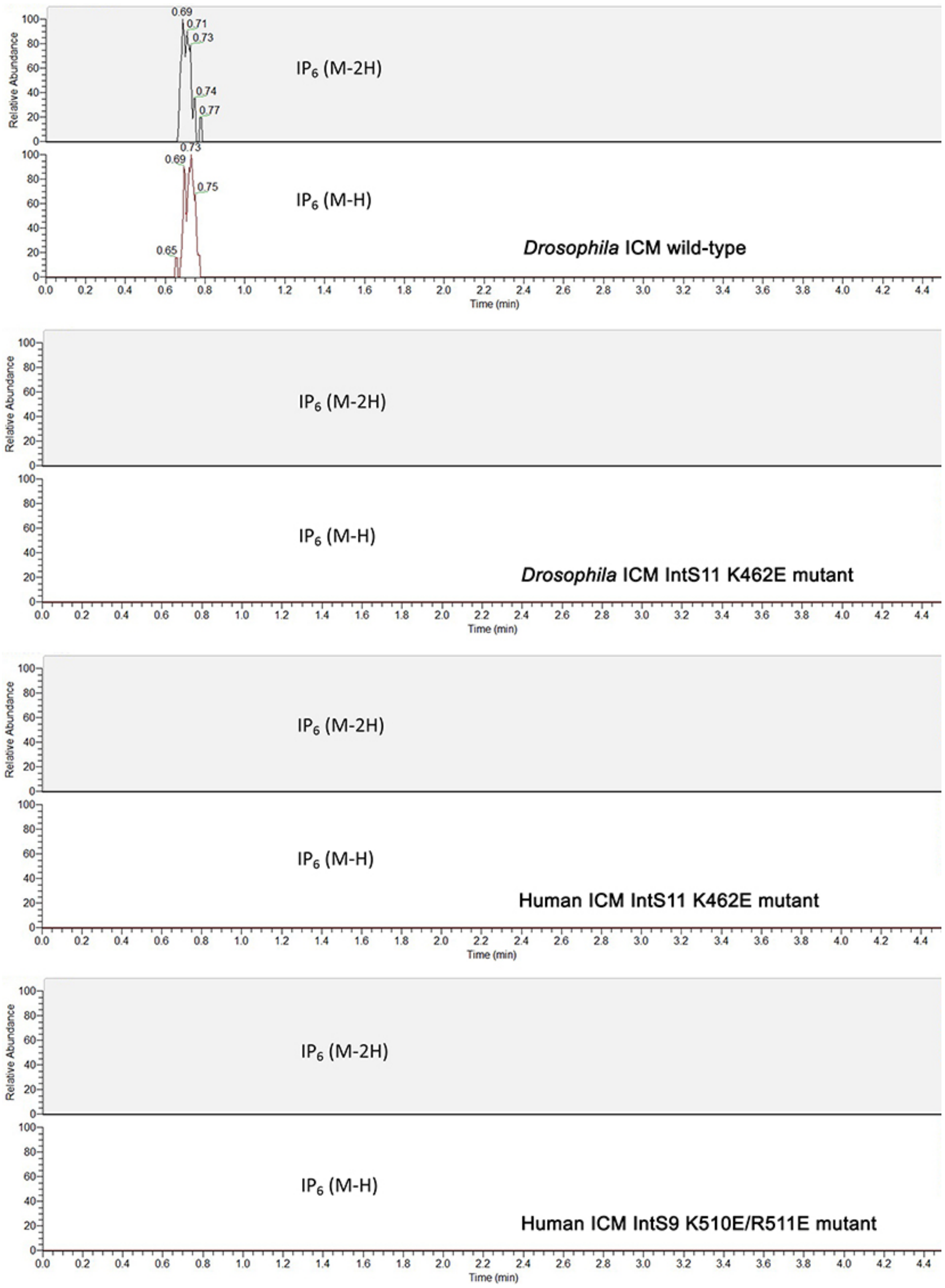

Supplementary Fig. 2. No IP_6 binding to mutant ICM samples was detected by mass spectrometry. LC-MS spectra of wild-type and mutant *Drosophila* ICM and mutant human ICM samples for [M-2H] and [M-H] ions of IP_6 are shown. While clear signals are observed for IP_6 with wild-type *Drosophila* ICM, no signals were observed for the mutant samples.

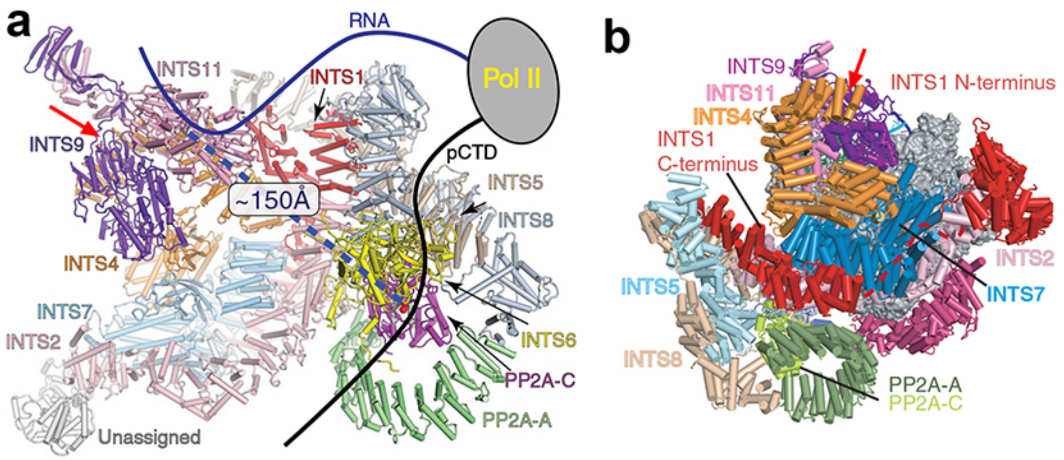

Supplementary Fig. 3. The IP_6 binding site is far away from other modules of Integrator and Pol II. **(A)**. The IP_6 binding site in the structure of human Integrator is indicated with the red arrow. Modified from Ref. 21. **(B)**. The IP_6 binding site in the structure of human Integrator-Pol II complex is indicated with the red arrow. Modified from Ref. 26.

Supplementary Table 1. Primer sequenes

Primer	5'>3' sequence
**Site-directed mutagenesis of INTS11**	
Drosophila K462E (F)	CCCTGCTTGAAGCAGAGGCC
Drosophila K462E (R)	GGCCTCTGCTTCAAGCAGGG
**Site-directed mutagenesis of INTS9**	
Drosophila R504D (F)	GGCGAGATCATAGATCTGCCATTAAAACGG
Drosophila R504D (R)	CCGTTTTAATGGCAGATCTATGATCTCGCC
Drosophila K508E (F)	CGTCTGCCATTAGAACGGAAGCTGG
Drosophila K508E (R)	CCAGCTTCCGTTCTAATGGCAGACG
Drosophila R509E (F)	CGTCTGCCATTAAAAGAGAAGCTGGATCG
Drosophila K509E (R)	CGATCCAGCTTCTCTTTTAATGGCAGACG
Drosophila R2E (F)	GGATCCAGAATTGTATTGTCTCAGCGGGGACC
Drosophila R2E (R)	GCGGCCGCCTAAAAACTCTGTAAGCATTTCATGATG[illegible]
**Primers used for luciferase cloning**	
KpnI Renilla luciferase (F)	GGCCGGTACCATGGCTTCCAAGGTGTACGA
MfeI Renilla luciferase (R)	GGCCCAATTGCGCCTAGAATTACTGCTCGT
**Drosophila qPCR amplicons**	
U4 snRNA misprocessing (F)	GGTGGCAATACCGTAACCAAT
U4 snRNA misprocessing (R)	GGCTAAGACAACCGTCATATTAA
Tiggrin (F)	GCAACATATGGGTCGGTGAC
Tiggrin (R)	CTTGTTGCGGATCAGGTAGC
Bj1 (F)	CGCAGAAAGGCCCTGACTAAT
Bj1 (R)	AGCTCCAGATGAAAAGCGATTC
