# Supplementary material for: Inositol hexakisphosphate is required for Integrator function
Source: Nat Commun. 2022 Sep 30;13:5742. doi: 10.1038/s41467-022-33506-3 (PMC9525679; doi:10.1038/s41467-022-33506-3)
Supplement: Supplementary file 2 — Reporting Summary [file 41467_2022_33506_MOESM2_ESM.pdf]

nature portfolio

Corresponding author(s): Liang Tong, Eric Wagner

Last updated by author(s): Aug 29, 2022

Reporting Summary

Nature Portfolio wishes to improve the reproducibility of the work that we publish. This form provides structure for consistency and transparency in reporting. For further information on Nature Portfolio policies, see our Editorial Policies and the Editorial Policy Checklist.

Statistics

For all statistical analyses, confirm that the following items are present in the figure legend, table legend, main text, or Methods section.

n/a	Confirmed	
☐	☒	The exact sample size (*n*) for each experimental group/condition, given as a discrete number and unit of measurement
☒	☐	A statement on whether measurements were taken from distinct samples or whether the same sample was measured repeatedly
☒	☐	The statistical test(s) used AND whether they are one- or two-sided<br>*Only common tests should be described solely by name; describe more complex techniques in the Methods section.*
☒	☐	A description of all covariates tested
☒	☐	A description of any assumptions or corrections, such as tests of normality and adjustment for multiple comparisons
☐	☒	A full description of the statistical parameters including central tendency (e.g. means) or other basic estimates (e.g. regression coefficient) AND variation (e.g. standard deviation) or associated estimates of uncertainty (e.g. confidence intervals)
☒	☐	For null hypothesis testing, the test statistic (e.g. *F*, *t*, *r*) with confidence intervals, effect sizes, degrees of freedom and *P* value noted<br>*Give P values as exact values whenever suitable.*
☒	☐	For Bayesian analysis, information on the choice of priors and Markov chain Monte Carlo settings
☒	☐	For hierarchical and complex designs, identification of the appropriate level for tests and full reporting of outcomes
☒	☐	Estimates of effect sizes (e.g. Cohen's *d*, Pearson's *r*), indicating how they were calculated

Our web collection on statistics for biologists contains articles on many of the points above.

Software and code

Policy information about availability of computer code

Data collection	Leginon 3.5
Data analysis	cryoSPARC 3.0.1, RELION 3.1, Coot 0.9.4, Chimera 1.14, PyMOL 1.8, PHENIX 1.13, Prism 9.0

For manuscripts utilizing custom algorithms or software that are central to the research but not yet described in published literature, software must be made available to editors and reviewers. We strongly encourage code deposition in a community repository (e.g. GitHub). See the Nature Portfolio guidelines for submitting code & software for further information.

Data

Policy information about availability of data

All manuscripts must include a data availability statement. This statement should provide the following information, where applicable:

- Accession codes, unique identifiers, or web links for publicly available datasets
- A description of any restrictions on data availability
- For clinical datasets or third party data, please ensure that the statement adheres to our policy

The structure of the Drosophila ICM-IP6 complex has been deposited at the PDB under accession code 7SN8 [http://doi.org/10.2210/pdb7SN8/pdb]. The cryo-EM map of the Drosophila ICM-IP6 complex has been deposited at the EMDB under accession code 25214 [https://www.ebi.ac.uk/emdb/entry/EMD-25214].

Human research participants

Policy information about studies involving human research participants and Sex and Gender in Research.

Reporting on sex and gender	N/A
Population characteristics	N/A
Recruitment	N/A
Ethics oversight	N/A

Note that full information on the approval of the study protocol must also be provided in the manuscript.

Field-specific reporting

Please select the one below that is the best fit for your research. If you are not sure, read the appropriate sections before making your selection.

☒ Life sciences ☐ Behavioural & social sciences ☐ Ecological, evolutionary & environmental sciences

For a reference copy of the document with all sections, see nature.com/documents/nr-reporting-summary-flat.pdf

Life sciences study design

All studies must disclose on these points even when the disclosure is negative.

Sample size	The experiments have been biologically triplicated for calculating/plotting of mean and standard deviation and thus no sample size calculation was performed. The difference of means obtained from each biologically triplicated sample compared are all greater than 2 standard deviation. It suggests that results are statistically significant.
Data exclusions	The results obtained in the study have been included
Replication	The experiments have been performed independently with biological triplicates.
Randomization	The samples have been assigned randomly at the beginning of experiments.
Blinding	The structure analyses have been corroborated with wet lab experiments.

Reporting for specific materials, systems and methods

We require information from authors about some types of materials, experimental systems and methods used in many studies. Here, indicate whether each material, system or method listed is relevant to your study. If you are not sure if a list item applies to your research, read the appropriate section before selecting a response.

Materials & experimental systems

n/a	Involved in the study	
☐	☒	Antibodies
☐	☒	Eukaryotic cell lines
☒	☐	Palaeontology and archaeology
☒	☐	Animals and other organisms
☒	☐	Clinical data
☒	☐	Dual use research of concern

Methods

n/a	Involved in the study	
☒	☐	ChIP-seq
☒	☐	Flow cytometry
☒	☐	MRI-based neuroimaging

Antibodies

Antibodies used	anti-hInts11 (Bethyl, #A301-274A), anti-hIPMK (Thermo, #PA5-21629), anti-GFP JL-8 clone(Clontech, #632381), anti-alpha Tubulin (abcam, #ab15246), and anti-GAPDH GA1R clone(Thermo, #MA5-15738), anti-FLAG-HRP conjugted M2 clone (Sigma, #A8592)
Validation	Anti-alpha-Tubulin polyclonal antibody is registered with ID: AB_301787. It has been validated by company on Western blot application. This antibody gives a predominant band at expected molecular weight around 55KD after blotting whole cell extracts from mammalian cell lines.

Anti-GAPDH monoclonal antibody (clone GA1R) is registered with ID: AB_437392. It has been validated by company on western blot application. This antibody gives a single band at expected molecular weight around 37KD after blotting whole cell extracts from mammalian cell lines.
Anti-Flag-HRP (M2 clone) monoclonal antibody is registered with ID: AB_439702. It is used for detection of Flag fusion proteins (N-terminal and C-terminal) on Western blots application. The minimum detection range of Flag-fusion protein tested by company is around 8ng shown on Dot blot.
Anti-GFP monoclonal antibody (clone JL-8) is registered with ID: AB_10013427. It is recommended by company to be used for western blot application to detect N- and C-terminal fusion protein containing this GFP in mammalian cell lysates.
Anti-hIPMK polyclonal antibody is registered with ID: AB_11152441. It has been validated by company on western blot application to detect hIPMK from mammalian cell lysates.
Anti-hInts11 polyclonal antibody is registered with ID: AB_937779. It has been validated by company on western blot application to detect human Ints11 from whole cell lysates. This antibody gives a distinct band at expected molecular weight around 67KD on blot.
The citations of each antibody can be found on the website, The Antibody Registry, by its ID.

Eukaryotic cell lines

Policy information about cell lines and Sex and Gender in Research

Cell line source(s)	293T cells (ATCC) , DL1 cells (DGRC) and Tni cells (Expression Systems)
Authentication	The 293T cell line was authenticated by STR profiling by ATCC. DL1 cells and the Tni cell line were not authenticated.
Mycoplasma contamination	The 293T and DL1 cell lines were tested negative of mycoplasma in our lab using MycoSensor qPCR Assay Kits (#302107, Agilent). The Tni cells were not tested for mycoplasma contamination.
Commonly misidentified lines (See ICLAC register)	There is no commonly misidentified cell lines for 293T or DL1 or Tni in this study.
