# Supplementary material for: Inositol hexakisphosphate is required for Integrator function
Source: Nat Commun. 2022 Sep 30;13:5742. doi: 10.1038/s41467-022-33506-3 (PMC9525679; doi:10.1038/s41467-022-33506-3)
Supplement: Supplementary file 3 — Source Data [file 41467_2022_33506_MOESM3_ESM.pdf]

Figure 6d

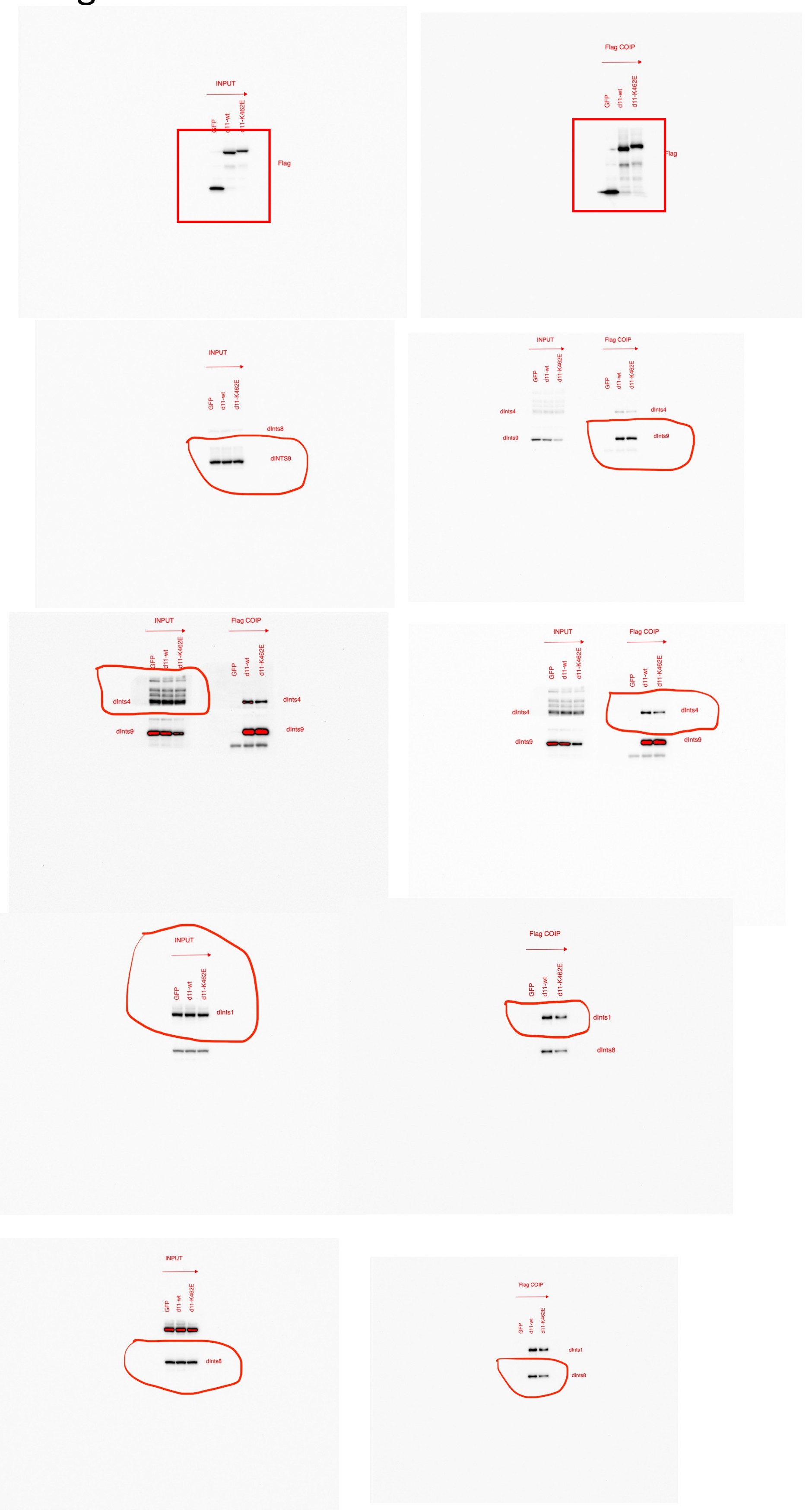

INPUT
GFP
d11-wt
d11-K462E
Flag
Flag COIP
GFP
d11-wt
d11-K462E
Flag
INPUT
GFP
d11-wt
d11-K462E
dInts8
dINTS9
INPUT
Flag COIP
GFP
d11-wt
d11-K462E
dInts4
dInts9
dInts4
dInts9
INPUT
Flag COIP
GFP
d11-wt
d11-K462E
dInts4
dInts9
dInts4
dInts9
INPUT
Flag COIP
GFP
d11-wt
d11-K462E
dInts4
dInts9
dInts4
dInts9
INPUT
GFP
d11-wt
d11-K462E
dInts1
Flag COIP
GFP
d11-wt
d11-K462E
dInts1
dInts8
INPUT
GFP
d11-wt
d11-K462E
dInts8
Flag COIP
GFP
d11-wt
d11-K462E
dInts1
dInts8

Figure 6e

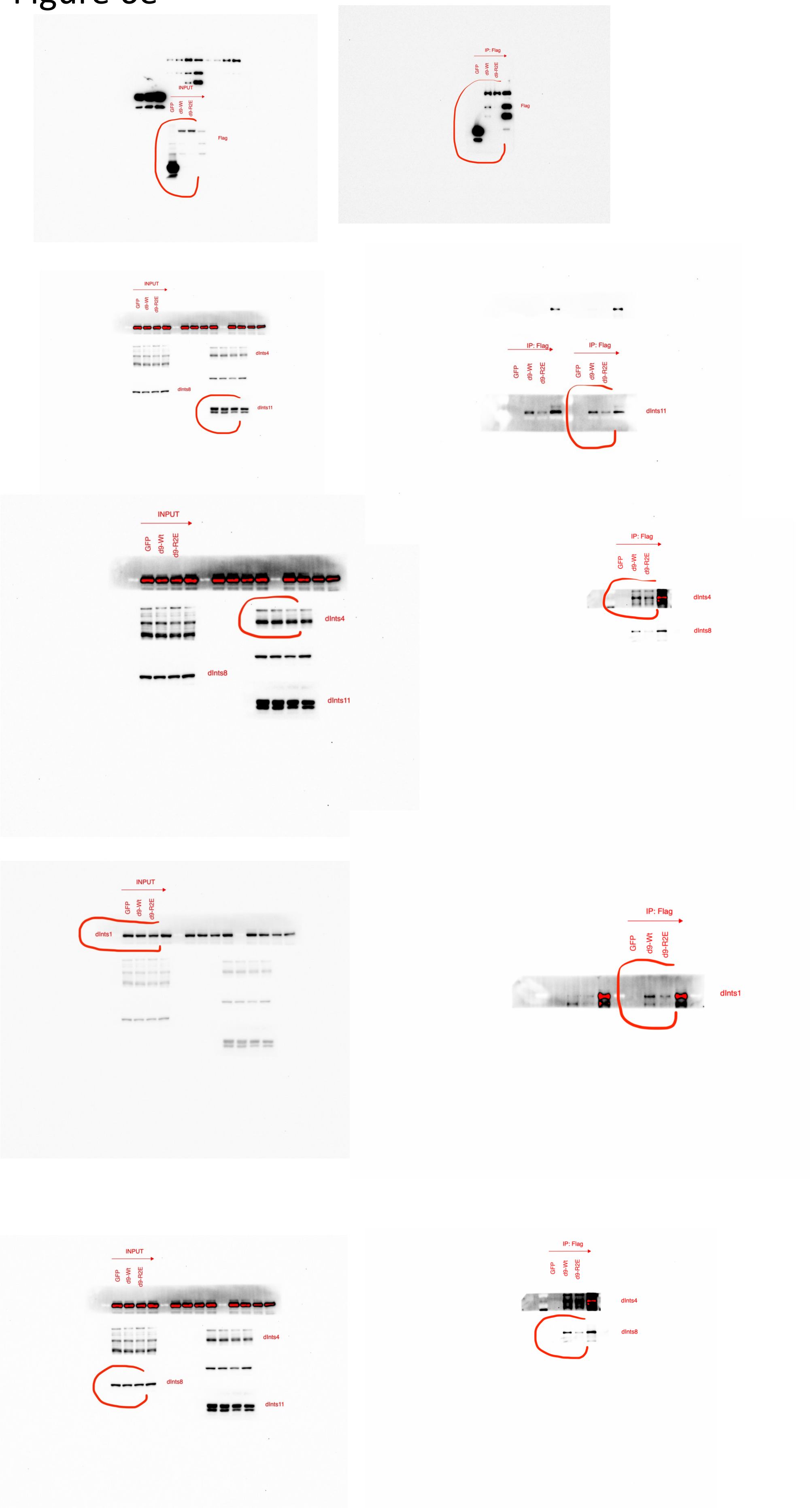
INPUT
GFP
d9-Wt
d9-R2E
Flag
IP: Flag
dInts4
dInts8
dInts11
dInts1

Figure 7a

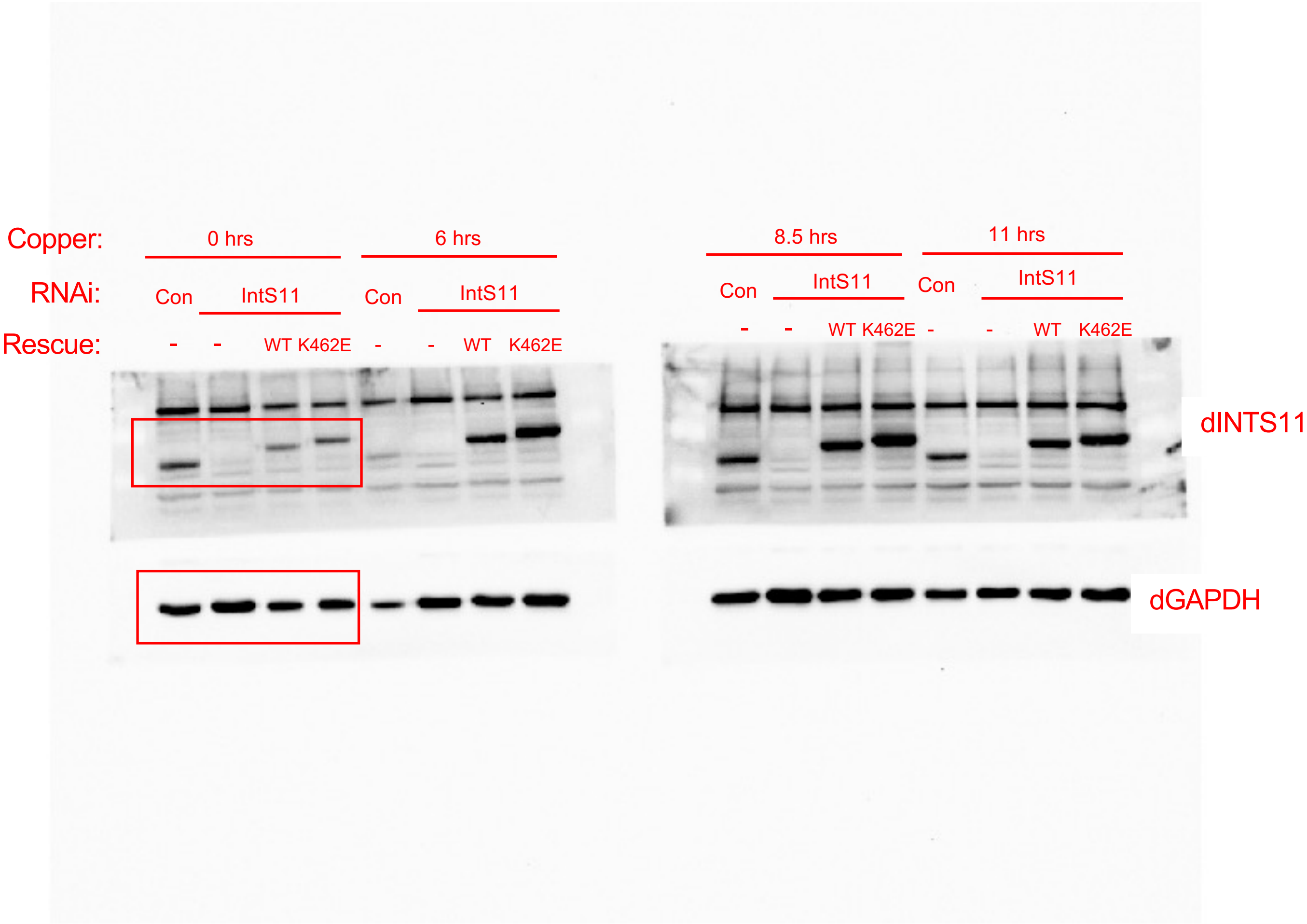

Figure 7b raw numbers

	tig			U4 mis			Bj1		
Control	0.94	0.88	1.21	0.94	0.89	1.19	0.91	1.03	1.07
+GFP	10.86	13.65	10.23	7.34	8.3	7.33	0.59	0.69	0.65
+11 WT	2.26	2.07	2.25	2.27	2.16	1.24	0.77	0.79	0.76
+11 K462E	8.23	9.33	6.77	7.61	7.09	6.18	0.68	0.68	0.74

Figure 7c

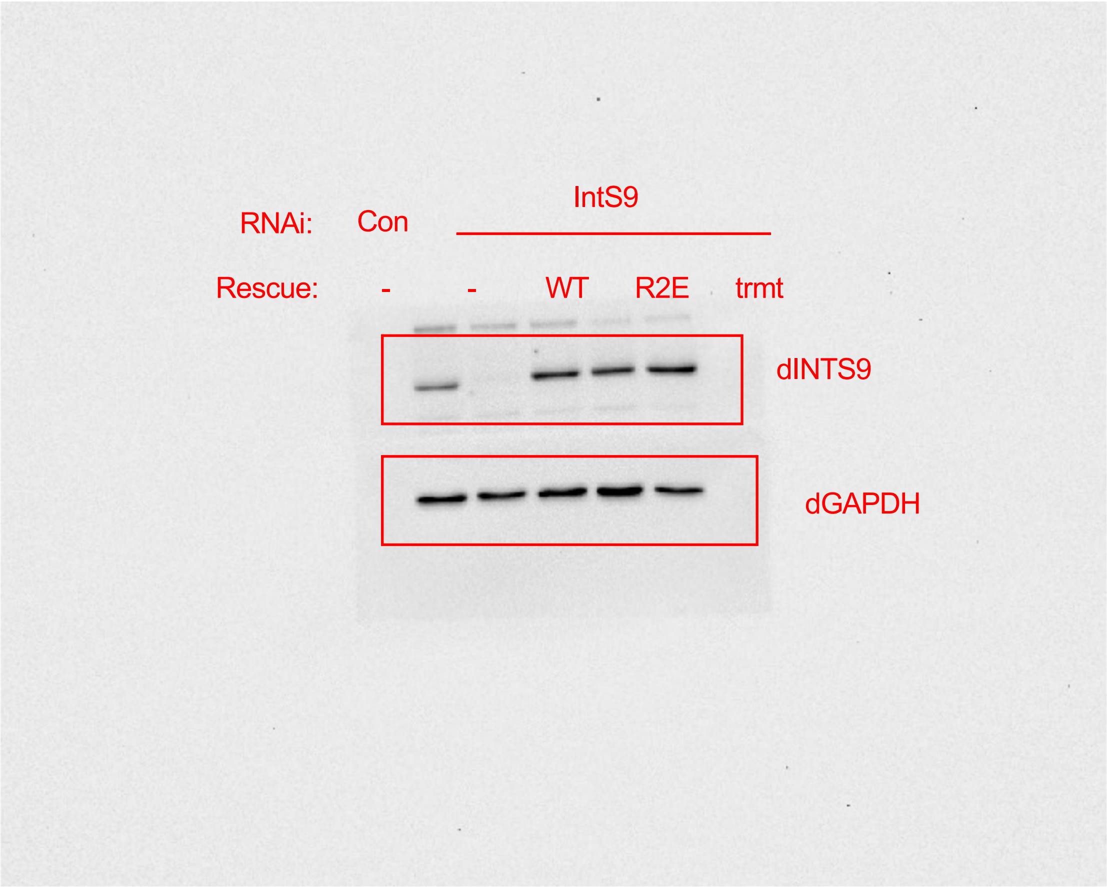

Figure 7d raw numbers

	tig			U4 mis			Bj1		
Control	0.91	0.98	1.13	0.67	1.22	1.23	1.0008	0.9987	1.0005
+ GFP	19.74	19.31	18.17	10.89	10.81	9.17	1.0003	1.1159	0.9416
+ 9wt	3.12	3.49	3.03	3.46	3.5	3.86	0.9965	0.9666	0.9314
+9R2E	19.59	20.41	20.28	9.61	8.47	9.73	1.1071	0.9939	0.9392
+9trmt.	20.67	19.13	21.96	11.02	11.03	13	0.9653	1.1004	0.9465

Figure 8b

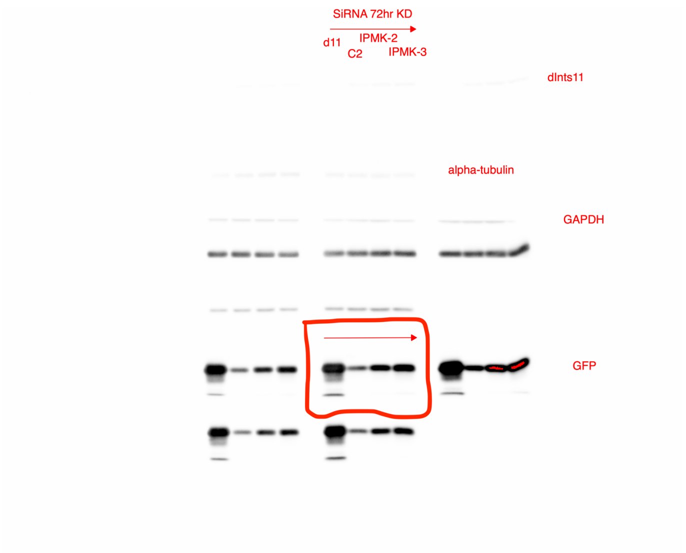
SiRNA 72hr KD
d11
IPMK-2
C2
IPMK-3
dInts11
alpha-tubulin
GAPDH
GFP

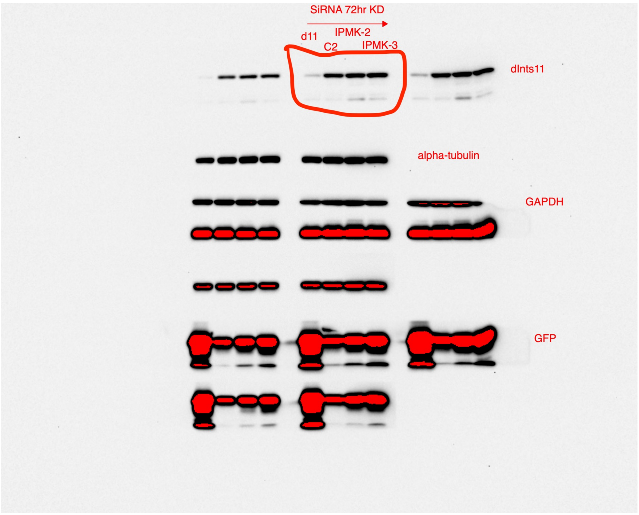
SiRNA 72hr KD
d11
IPMK-2
C2
IPMK-3
dInts11
alpha-tubulin
GAPDH
GFP

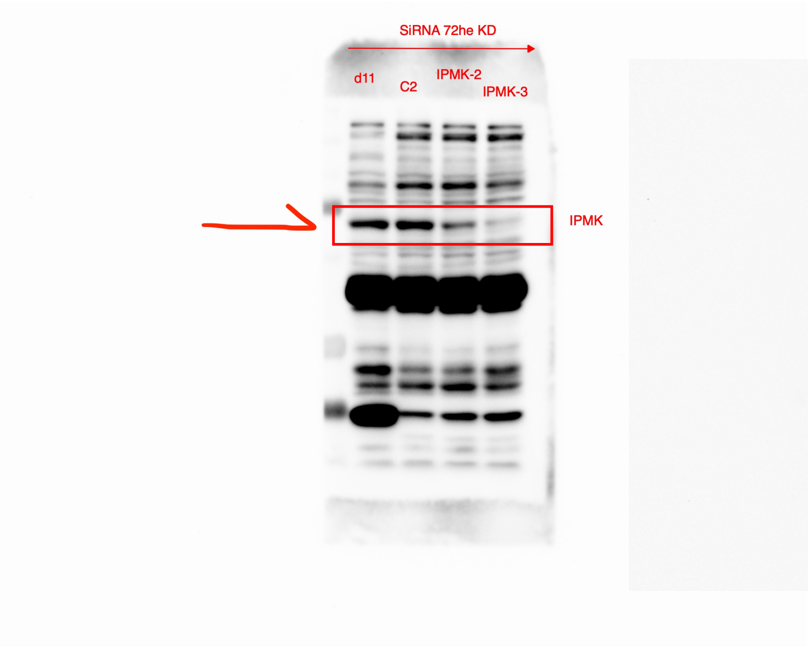
SiRNA 72he KD
d11
C2
IPMK-2
IPMK-3
IPMK

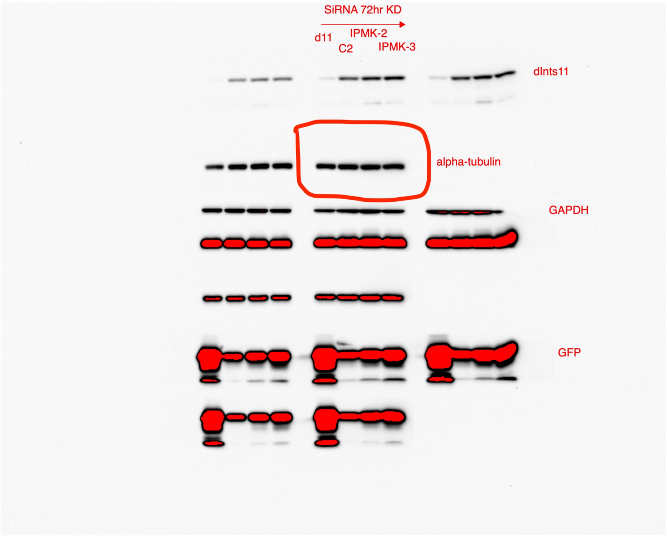
SiRNA 72hr KD
d11
IPMK-2
C2
IPMK-3
dInts11
alpha-tubulin
GAPDH
GFP

Figure 8c

FH 21-5 & 21-8

	A	B	C	D	E	F	G	H	I	J
1										
2	parental U11-Rluc #13 (same amount of cells from 24 well, but diluted into various amount of lysis buffer)									
3	Lysis buffer	OD595	Read1	Read2						
4	400	0.45	599	481		bradford measurement (600ul bradford and 2ul sample)				
5	200	0.542	1227	968		Luciferase measurement (100ul reagent and 6ul sample)				
6	100	0.668	1921	1457						
7	50	0.857	2806	1797						
8										
9	Experiments (each well lysed in 200ul of buffer)				Background		Read1 (Background correc		Read1 averag	Read1 SD
10	C2-1	0.485	912	623	865.103		46.897		62.8722	19.317179
11	C2-2	0.506	1062	845	977.6588		84.3412			
12	C2-3	0.492	960	765	902.6216		57.3784			
13	Si11-1	0.504	4274	3377	966.9392		3307.0608		3376.3536	232.57613
14	Si11-2	0.495	4105	3026	918.701		3186.299			
15	Si11-3	0.505	4608	3646	972.299		3635.701			
16	SiIPMK2-1	0.527	1896	1503	1090.2146		805.7854		994.50667	165.65909
17	SiIPMK2-2	0.538	2211	1750	1149.1724		1061.8276			
18	SiIPMK2-3	0.535	2249	1793	1133.093		1115.907			
19	SiIPMK3-1	0.53	3262	2674	1106.294		2155.706		2521.2792	316.6057
20	SiIPMK3-2	0.522	3770	2955	1063.4156		2706.5844			
21	SiIPMK3-3	0.536	3840	3179	1138.4528		2701.5472			
22										
23		Read1 averag	Read1 SD							
24	C2-1	62.8722	19.317179							
25	Si11-1	3376.3536	232.57613							
26	SiIPMK2-1	994.50667	165.65909							
27	SiIPMK3-1	2521.2792	316.6057							

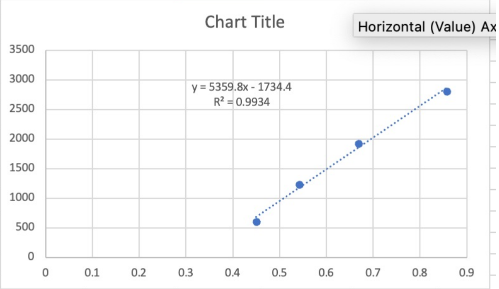

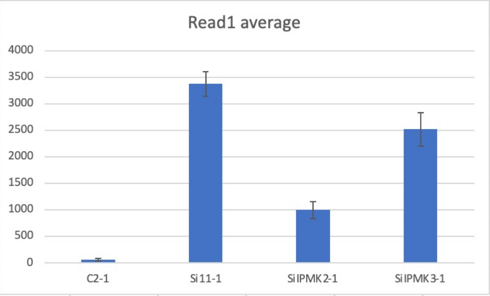

FH 21-8 FH 21-5
